# Isolation and Identification of Constituents Exhibiting Antioxidant, Antibacterial, and Antihyperuricemia Activities in *Piper methysticum* Root

**DOI:** 10.3390/foods11233889

**Published:** 2022-12-01

**Authors:** Truong Ngoc Minh, Truong Mai Van, Tran Dang Khanh, Tran Dang Xuan

**Affiliations:** 1 Center for Research and Technology Transfer, Vietnam Academy of Science and Technology (VAST), Hanoi 122100, Vietnam; 2 Transdisciplinary Science and Engineering Program, Graduate School of Advanced Science and Engineering, Hiroshima University, Hiroshima 739-8529, Japan; 3 Agricultural Genetic Institute, Pham Van Dong Street, Hanoi 122000, Vietnam; 4 Center for Agricultural Innovation, Vietnam National University of Agriculture, Hanoi 131000, Vietnam; 5 Center for the Planetary Health and Innovation Science (PHIS), The IDEC Institute, Hiroshima University, Higashi-Hiroshima 739-8529, Japan

**Keywords:** *Piper methysticum* L., antioxidant activity, antibacterial activity, flavonoid, kavalactone, beverage

## Abstract

The aqueous extract of kava (*Piper methysticum*) root is known as a traditional beverage for daily intake in the Western Pacific Islands, such as Fiji, Tonga, and Vanuatu, to induce relaxation and health-beneficial effects. In this study, the antioxidant, anti-hyperuricemia, and antibacterial properties of kava root were investigated through the isolation and purification of bioactive compounds in ten fractions separated by column chromatography (CC). They included six flavonoids, 5-hydroxy-4′,7-dimethoxyflavanone (**C1**), matteucinol (**C2**), isosakuranetin (**C3**), 5,7- dimethoxyflavanone (**C4**), 2′,4′-dihydroxy-6′-methoxydihydrochalcone (in **MC5**) and alpinetin (**C10**), and seven kavalactones, 5,6-dehydrokawain (DK) (in **MC5** and **C6**), kavain (in **MC7**), yangonin (in **MC7** and **C8**), dihydro-5,6-dehydrokavain (DDK) (in **MC9**), 7,8-dihydromethysticin (in **MC9**), dihydromethysticin (in **MC9**), methysticin (in **MC9**). The chemical structures of the compounds were illustrated by the analyses of gas chromatography–mass spectrometry (GC–MS), electrospray ionization–mass spectrometry (ESI–MS), nuclear magnetic resonance (^1^H and ^13^C-NMR), and X-ray diffraction. The evaluation of the free radical scavenging activity of the isolated substances via the DPPH and ABTS assays revealed that **C3** (IC_50_: ABTS = 76.5; DPPH = 74.8 µg/mL) possessed the strongest antioxidant property. In terms of anti-hyperuricemia activity evaluated via the xanthine oxidase inhibitory in vitro assay, the compound **C10** was the most promising inhibitor, revealing an IC_50_ of 134.52 µg/mL. The two kavalactone mixtures in **MC5** and a pure compound **C6** inhibited the growth of bacteria *Listeria monocytogenes*, while **MC7** can constrain the development of *Klebsiella pneumoniae.* This is the first study to isolate, purify, and identify the flavonoids isosakuranetin, 2′,4′-dihydroxy-6′-methoxydihydrochalcone and alpinetin in kava root and report their pharmaceutical potential. The identified bioactive compounds showed potent antioxidant, anti-hyperuricemia, and antibacterial activity and thus can enhance the value of beverages and foods derived from kava root.

## 1. Introduction

Free radicals are described as highly reactive and unstable molecules, which is caused by an unpaired electron [1,2]. They are generated either from the normal cellular metabolism process or from external sources including pollution, cigarette smoke, radiation, and medication. The overproduction of free radicals and reactive oxygen-containing molecules in the human body affects important macromolecules, which then causes significant damage to cell structures and disrupts the homeostatic balance [3,4]. This further leads to a wide range of chronic and degenerative diseases, such as cancer, cardiovascular diseases, inflammation, diabetes, and neurodegeneration [5]. Consequently, antioxidant and antibacterial compounds isolated from medicinal plants gain particular interest as they are potential agents in neutralizing the adverse effects of harmful free radicals and enhancing the oxidative stability [6,7,8]. They can be effectively used in food additives, cosmetic products, and pharmaceutical drugs [8,9].

Xanthine oxidase (XO) is an important biological generator of oxygen free radicals. This is a key enzyme involved in the metabolism of hypoxanthine to xanthine, which was successively converted into uric acid by the oxidation process [10,11,12,13]. The excessive production of uric acid in blood results in a condition known as hyperuricemia. This further leads to the formation of the monosodium urate crystal and causes painful gouty inflammation [5]. Recently, there has been an increase in the prevalence of hyperuricemia worldwide [5,14,15]. To date, only three synthetic XO inhibitors, i.e., allopurinol, febuxostat and oxypurinol, have been approved as hyperuricemia treatments and anti-gout agents [16]. However, their side effects, such as skin rash, allergic reactions and kidney failure, have been highlighted. As a result, nature-based XO inhibitors are more preferable and need further investigations of their safety and efficiency.

Kava (*Piper methysticum*), a member of the pepper family, is an exclusive perennial plant found on the Pacific Islands. Due to the effect of relaxation, resulting in the enhancement of social ties and conflict avoidance, the aqueous extract from kava is a preferred beverage during important events or community gatherings on several islands, such as Fiji, Tonga and Vanuatu [17]. This herbal plant also holds a significant place in the local residents’ daily intake, as it has many health benefits [18,19,20]. Kava root has traditionally been used by indigenous people as an effective folk remedy for a variety of diseases, such as gonorrhea, menstrual pain, tuberculosis, arthritic conditions, and chronic gout [21,22,23]. Kava consumption has also increased, and it has become popular in new regions, including Australia, Europe and North America, where it is sold at medicinal and recreational markets [24]. The various chemical compositions of kava have been reported in a number of studies, including more than 40 compounds within the classes of kavapyrones, alkaloids, steroids, chalcones, long-chained fatty acids and alcohols [18]. Among them, a group of components known as kava pyrones or kava lactones is believed to be responsible for the therapeutic effects [18,25]. Although the pharmaceutical benefits of kava in treating inflammatory arthritis were described above, no research on phytochemicals attributed to this property has been conducted so far. To answer the question of whether other compounds besides kava lactones are associated with the anti-gout and hyperuricemia effects, research on further bioactive components is needed.

In this work, the isolation and purification of the active compounds, employing column chromatography (CC), was performed, followed by the identification of its bioactivity, including in vitro assays of XO inhibitory, antioxidant and antibacterial activity. The chemical structures of the isolated second metabolites were illustrated by gas chromatography–mass spectrometry (GC–MS), electrospray ionization–mass spectrometry (ESI–MS), nuclear magnetic resonance (^1^H and ^13^C-NMR) and X-ray diffraction analysis. Therefore, the additional value of beverages and foods derived from kava root is highlighted.

## 2. Materials and Methods

### 2.1. Plant Material

The dried root part of commercial kava plant was obtained from Kava King Products, Inc. (Orchard St, Ormond Beach, FL, USA) on 2 May 2017. The powder sample was stored at −20 °C in the freezer prior to experiments.

### 2.2. Isolation and Purification Procedure of Antioxidant Compounds

**Figure 1:**
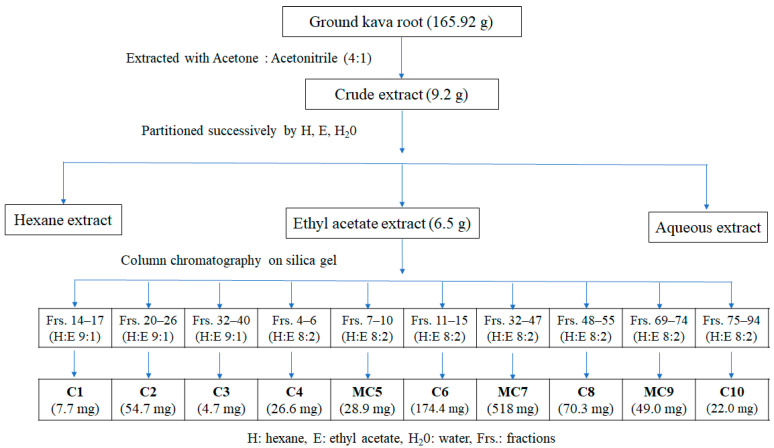
Fractionation and column chromatography separation of kava root.

The isolation process of bioactive compounds in kava root was reported by Van et al. [26]. The ground kava root (165.9 g) was first immersed in acetone–acetonitrile (4:1) at room temperature for one week. The filtrate was obtained with filter paper (Whatman No. 5C, 110 mm), followed by evaporation using a rotary evaporator (SB–350–EYELA, Tokyo Rikakikai Co., Ltd., Tokyo, Japan) at 45 °C to yield 9.4 g crude extract. After this, the crude extract was dissolved in distilled water, and then extracted successively with hexane and EtOAc. The crude extract (6.5 g) obtained by evaporating the EtOAc layer was further chromatographed into a column ((20 mm diameter × 500 mm height, Climbing G2, Tokyo, Japan) over a silica gel (size Ǻ 60, 200–400 mesh particle size, Sigma-Aldrich, Darmstadt, Germany) and eluted with a gradient of n-hexane–EtOAc (10:0–0:10 *v*/*v*). The fractions were collected separately (frs.; each of 100 mL) and concentrated under a rotary evaporator. This was followed by inspection via thin layer chromatography (TLC) to verify the purity of compounds and determine the number of components. The recorded similar spots on TLC sheets were combined. Fractions were then crystallized at room temperature to give thirteen substances, as described in Figure 1. Compounds **C1**, **C2**, **C3**, **C4**, **C6**, **C8** and **C10** were purified after elution by hexane, followed by rapid filtering through filter paper to remove traces of impurities. The yields of the 10 fractions were 7.7, 54.7, 4.7, 26.6, 28.9, 174.4, 518, 70.3, 49.0 and 22.0 mg, respectively (Figure 1). The chemical formulas of isolated substances were determined by gas chromatography–mass spectrometry (GC–MS), electrospray ionization–mass spectrometry (ESI–MS) and X-ray analysis. The new compounds with high purity levels were further subjected to either ^1^H and ^13^C nuclear magnetic resonance (NMR) or X-ray analysis for the elucidation of their molecular structures (Supplementary Table S1).

### 2.3. Identification of Antioxidative Compounds

The active compounds and mixtures were analyzed by a GC–MS machine (JMS-T100 GCV, JEOL Ltd., Tokyo, Japan) equipped with a DB-5MS capillary column, 30 m in length, with a 0.25 mm internal diameter and 0.25 µm in thickness (Agilent Technologies, J & W Scientific Products, Folsom, CA, USA). The column temperature was set initially at 50 °C without a hold time, followed by an increase to 300 °C with the gradient of 10 °C/min and held for 20 min. The temperatures of the injector and detector were programmed, respectively, at 300 °C and 320 °C, with a mass scan range of 29–800 amu. The compounds were determined via a comparison of their mass spectral fragmentation patterns with the mass spectral libraries of JEOL’s GC-MS Mass Center System Version 2.65a. The compounds with high purity were subjected to further spectroscopic techniques for structure elucidation.

ESI/MS analysis was implemented using a mass spectrometer with an electrospray ion source. The measurements were conducted in the positive mode with an ion spray voltage of 3000 V and a capillary temperature of 350 °C. The mass spectra were recorded in full scan mode within the range of 280 to 1000 m/z.

The nuclear magnetic resonance spectroscopy (^1^H (400.13 Hz) and ^13^C (100.612 Hz)) were recorded using a Bruker 500 model spectrometer (BRUKER BioSpin, Faellanden, Switzerland). The pure compounds were dissolved in chloroform (CDCl_3_) to obtain a concentration of 3000 ppm (µg/mL). The spectral characteristics of compounds were compared with those in the literature to elucidate the chemical structures.

The X-ray diffraction pattern was recorded using a single-crystal X-ray diffractometer from Bruker, connected with a highly sensitive ApexII CCD area detector. The single crystal was packed tightly in the sample holders and freeze-dried at the temperature of −100 °C. The average wavelength was 0.71073 Å. The obtained X-ray powder diffraction patterns of the samples were compared with a database from the website of the International Union of Crystallography. Finally, the compound structures of the samples were reported in a crystallographic information file (CIF).

### 2.4. Bioactivity Assays

#### 2.4.1. Antioxidant Assays

##### DPPH Radical Scavenging Assay

The 2,2-diphenyl-1-picrylhydrazyl (DPPH) radical scavenging activity assay was conducted according to the method described previously by Minh et al. [27], with minor modification. The various polarity extracts were evaluated at concentrations of 500, 1000, 2000 and 3000 ppm, while the content of isolated compounds was 50, 100, 500, 1000 and 1500 ppm. A volume of 100 µL of each sample was mixed with 50 µL DPPH 0.5 mM, followed by the addition of 100 µL sodium acetate buffer (pH 5.5, 0.1 M). In this study, BHT and methanol (MeOH) were used as a positive standard and control, respectively. After incubation in the dark for 30 min at room temperature, the mixture’s absorbance (Abs) was measured at 517 nm using a UV–VIS spectrophotometer (Multiskan GO; Thermo Fisher Scientific, Vantaa, Finland). This assay was implemented in triplicate and the antioxidant activity was calculated with the following equation:

DPPH radical scavenging activity (%) = [(Abs_control_ – Abs_sample_)/Abs_control_] × 100 (1)

where Abs_control_ is the absorbance of the control reaction, and Abs_sample_ is the absorbance of the test substance. Based on the value of DPPH radical scavenging activity, the IC_50_ value was calculated to evaluate the antioxidant ability of the tested samples. A smaller value of IC_50_ is associated with stronger antioxidant properties.

##### ABTS Radical Scavenging Assay

The ABTS radical scavenging capacity was determined by the ABTS cation decolorization assay, as described by Tuyen et al. [28]. The preparation of samples was in the same manner as the DPPH assay. The ABTS^+^ radical cation was produced by reacting 15 mL aqueous solution of ABTS 7 mM with 240 µL potassium persulfate at 140 mL. The mixture was kept in the dark at room temperature for 16 h before use. Prior to the assay, the ABTS^+^ was diluted with MeOH to achieve absorbance of 0.70 ± 0.05 at 734 nm. The reaction mixture contained 1 mL of prepared ABTS^+^ solution and 0.12 mL of each sample was measured after a 30-min incubation period in the dark at room temperature. The ABTS radical scavenging activity was expressed as in the following equation:

ABTS radical scavenging activity (%) = [(Abs_control_ – Abs_sample_)/Abs_control_] × 100 (2)

where Abs_control_ is the absorbance of the ABTS radical + methanol; Abs_sample_ is the absorbance of the ABTS radical + sample extract/standard.

#### 2.4.2. Xanthine Oxidase Inhibitory Activity

The inhibitory effect on xanthine oxidase of different extracts and isolated compounds was quantitated spectrophotometrically according to the method reported previously [29], with minor modifications. In this assay, allopurinol (6.25, 12.5, 25, 50 µg/µL) was used as a reference standard. The mixture consisting of 50 µL sample solution (3000, 2000, 1000, 500, 250, 125 and 67.5 ppm), 35 µL phosphate buffer (70 mM, pH = 7.5) and 30 µL fresh enzyme solution (0.01 units/mL in the same buffer) was prepared immediately before use. After pre-incubation at 25 °C for 15 min, the reaction was initiated by adding 60 µL of substrate solution (150 µM xanthine in the same buffer). The mixture was subsequently incubated at 25 °C for 30 min. The reaction was stopped by adding 25 µL HCl (1 M) and the absorbance was recorded at 290 nm using a microplate reader, as described above. A blank was prepared in the same manner, but the enzyme solution was added after pipetting HCl. XO inhibitory activity was expressed as the percentage inhibition of XO and calculated as follows:

$$\%\;\mathrm{Inhibition}=\{\frac{\left(A-B\right)-\left(C-D\right)}{\left(A-B\right)}\}\;\times \;100$$

where A is the activity of the enzyme without tested samples, and B is the control of A with neither tested samples nor enzyme. C and D denote the activity of the test solutions with and without XO, respectively. The IC_50_ value was calculated from the mean values of the data.

#### 2.4.3. Antibacterial Assay

The antibacterial activity of kava root was tested against five types of bacterial strains, namely *Pseudomonas aeruginosa, Escherichia coli*, *Klebsiella pneumoniae*, *Listeria monocytogenes* and *Bacillus subtilis.* This test was performed following a disc dilution method reported by Elzaawely et al. [30], with minor modifications. To begin with, 9-cm-diameter Petri dishes were coated with a 15 mL layer of nutrient agar, which was allowed to solidify. One hundred microliters of test organisms (10^6^ colony forming units (CFU)/mL)), after being cultured in nutrient broth media for 24 h, was laid evenly over the surface of the agar plate. A volume of 20 microliters of each sample, impregnated in a sterile filter paper disk (6 mm in diameter), was placed on the surface of the Petri dish. Ampicillin and streptomycin were used in this experiment as positive controls, and MeOH was utilized as a negative control. After 24 h incubation at 37 °C, bacterial inhibition was determined by measuring the diameter of the inhibition zone in millimeters, followed by comparison with standards and controls.

### 2.5. Statistical Analysis

The statistical analysis was performed by one-way ANOVA using Minitab^®^ 16.2.3 (copyright © 2022 Minitab Inc., Philadelphia, PA, USA). The results were reported as mean ± standard deviation values. Differences among treatment, control and standard data are considered significant at *p* < 0.05 using Tukey’s test.

## 3. Results

### 3.1. Phytochemical Isolation and Structure Elucidation

The most active EtOAc extract of kava root was subjected to CC and yielded ten fractions. The combination of hexane (H) and ethyl acetate (E) at ratios 9:1, 8:2 and 7:3 was the best method to yield secondary metabolites from kava root. Among them, H:E 8:2 was the most efficient elution to afford kava lactones.

**Figure 2:**
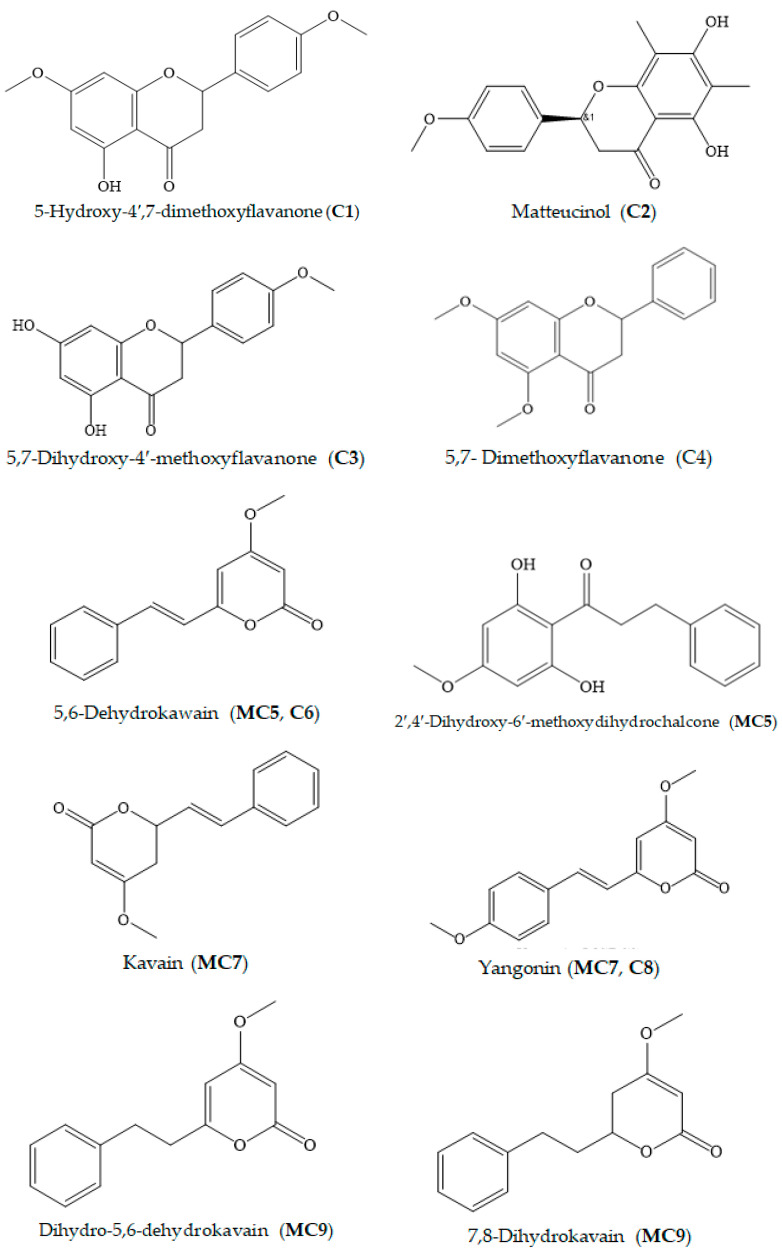

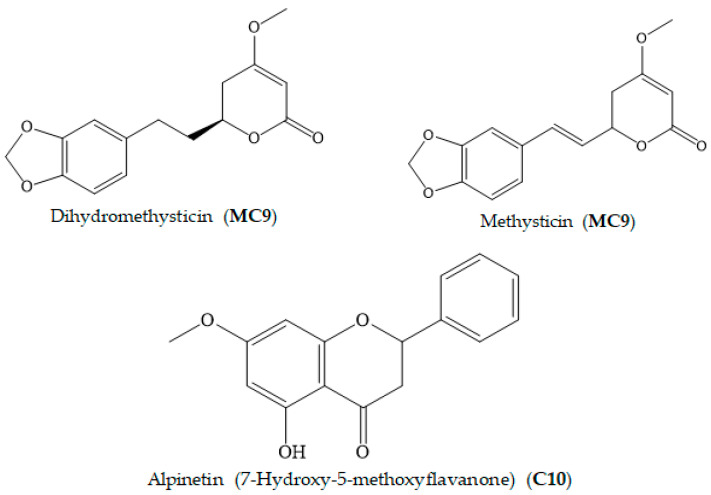
Chemical structures of components isolated from *P. methysticum* root.

The isolated metabolites were characterized and identified by GC–MS, ESI–MS, ^1^H and ^13^C NMR and X-ray analyses. The structure elucidation of compounds **C1**, **C2**, **C6**, **MC7**, **C8** and **MC9** was described previously in Van et al. 2018 [26]. The chemical structures of **C3** and **C4** were determined based on spectroscopic analyses and compared with previous literature data (Supplementary Data Figures S1–S6, Table S1). The X-ray technique was applied to identify the structures of 2′,4′-dihydroxy-6′-methoxydihydrochalcone and alpinetin. The obtained constituents belonged to the following groups. Flavanones: 5-hydroxy-4′,7-dimethoxyflavanone (**C1**), matteucinol (**C2**), 5,7-dihydroxy-4′-methoxyflavone (isosakuranetin, **C3**), 5,7- dimethoxyflavanone (**C4**) and alpinetin (**C10**). Kavalactones: DK (**MC5** and **C6**), kavain (**MC7**), yangonin (**MC7**, **C8**), DDK (**MC9**), 7,8-dihydromethysticin (**MC9**), dihydromethysticin (**MC9**) and methysticin (**MC9**). Chalcones: 2′,4′-dihydroxy-6′-methoxydihydrochalcone (**MC5**) (Table 1, Figure 2, Supplementary Data Figures S1–S10, CIF1-2). Among the isolated components, the compounds isosakuranetin (**C3**), isosakuranetin (**MC5**) and alpinetin (**C10**) were first identified from kava root.

The spectroscopic results of **C3** were interpreted as follows: compound **3**, obtained as yellow crystals, has molecular formula C_16_H_15_O_5_, determined by ESI-HRMS at 287.09131 (M+H)^+^ (calcd. 287.09140). The ^1^H and ^13^C NMR spectrum of **C3** displayed a typical flavanone-type peak pattern. The signal’s characteristic ^1^H NMR spectrum of **C3**, 5,7-dihydroxy-4′-methoxyflavanone, was observed at dH 12.2 (OH chelated), 7.39–7.46 (5H, m) for aromatic protons, and peak combination from dH 2.8, 3.1 and 5.4 could be assigned for the saturated of C-ring of the flavonoid. The B-ring was *p*-substituted by a phenyl group since both of the signal intensities at dC 128.9 and 126.1 were two-fold higher compared to others based on the signal pattern of the ^13^C NMR spectrum of **C3** (Supplementary Table S1).

### 3.2. Biological Activity of Crude Extracts

#### 3.2.1. Xanthine Oxidase Inhibitory (XOI) and Antioxidant Activity of Crude Extracts

The various extracts of kava root were investigated for XOI and free radical scavenging capacity using in vitro assays. The results, expressed as IC_50_ values, are summarized in Table 2. The IC_50_ was used as a parameter; a lower IC_50_ value represents a stronger capacity.

Among the studied extracts, the EtOAc extract exhibited the strongest inhibitory effects on both antioxidant and XOI activity. In the radical scavenging assays, the IC_50_ values of the EtOAc extract were 946 and 1007 µg/mL, as measured by the DPPH and ABTS methods. The antioxidant property of tested extracts decreased in the following order: EtOAc > water > chloroform > hexane. Meanwhile, the order of XOI activity was EtOAc > chloroform > hexane > water. The greatest inhibitory activity of the EtOAc extract revealed that it contained the most abundant active compounds. Therefore, this fraction was chosen for isolation and purification.

#### 3.2.2. Antibacterial Activity of Crude Extracts

The antibacterial activity of the kava root extracts against five human pathogenic bacteria was examined using the agar diffusion method. Ampicillin and streptomycin were used as standards and MeOH was used as a control. The corresponding zones of inhibition diameters are presented in Table 3, in which a higher value shows a stronger growth reduction. In all treatments, ampicillin and streptomycin demonstrated significantly higher suppression against the five studied bacteria compared to tested samples.

The result of a preliminary screening of the extracts indicated that although no extract could inhibit all tested species, each specific extract had a negative influence on the growth of a certain type of bacteria. The polar extract of EtOAc exhibited strong antibacterial activity against the *E. coli* strain; its zone of growth inhibition was 23 mm. The *B. subtilis* bacterial strain was observed as the most sensitive bacterium to the chloroform extract, with an inhibition zone of 15 mm. The non-polar hexane extract showed a potential effect against *P. mirabilis* as the inhibition zone diameter was 10 mm. Among the tested bacteria, *K. pneumoniae* and *L. monocytogenes* showed the strongest resistance against all extracts from kava root.

### 3.3. Biological Activity of Isolated Compounds

#### 3.3.1. Antioxidant Activity of Isolated Compounds

The antioxidant activity of the isolated constituents, estimated by the ABTS and DPPH methods, is presented in Table 4. The results varied among the tested compounds and studied assays. All isolated compounds except **MC7** were effective in scavenging ABTS free radicals. The ABTS scavenging activity of four fractions followed the order **C3** > **C4** > **C2** > **MC5** > **C1 > C6**. Meanwhile, the compounds **C3**, **C4** and **C6** were able to reduce the stable DPPH radicals and their antioxidant capacity can be ranked as follows: **C3** > **C4** > **C6**. Among six fractions isolated from the EtOAc extract, **C3** showed the highest inhibitory potential against ABTS and DPPH free radicals, with similar IC_50_ results (76.5 and 74.8 µg/mL, respectively). By contrast, **MC7**, **C8**, **MC9** and **C10** exhibited negligible antioxidant activity.

#### 3.3.2. XOI Activity of Isolated Compounds

The fractions **C4**, **MC5**, **C6**, **MC7**, **C8**, **MC9** and **C10** of kava root were examined for their inhibitory effects on the uric acid production from xanthine and xanthine oxidase (Table 5). The other fractions, **C1**–**C3**, with limited quantities, were not tested for this assay. The results showed that most of the tested fractions exhibited an XOI capacity that was comparable to that of allopurinol. The compound **C10** was the most promising XO inhibitor (IC_50_ = 134 µg/mL), followed by **MC7** and **C4** (IC_50_ = 242 and 268 µg/mL, respectively). The fractions **C6** and **MC9** displayed moderate inhibition against the activity of XO, with IC_50_ values of 452 and 465 µg/mL, respectively. Among the studied fractions, only compound **C8** showed trivial inhibition (Table 5).

### 3.4. Antibacterial Activity

In this study, several compounds isolated from the EtOAc extract were found to be responsible for the antibacterial activity of kava root (Table 6). The antibacterial activity of compounds **C1**–**C4** was not examined because their quantity constraints were not sufficient for the antibacterial experiment. Compound **MC5** was the most toxic compound against the *L. monocytogenes* strain, followed by **C6**. Their zones of inhibition were 13 mm and 9 mm, respectively. Compound **MC7** was found to be only potent as an antibacterial agent against *K. pneumoniae*, as it showed a 9.67 mm zone of inhibition. Two Gram-negative bacteria, i.e., *E. coli* and *P. mirabilis*, and one Gram-positive bacterium, *B. subtilis*, were not susceptible to any of the studied compounds.

## 4. Discussion

Flavonoids are a large class of natural products characterized by a flavone ring structure [31,32,33]. Flavonoid compounds are well known to possess potential inhibitory action towards free radicals and the XO enzyme, which is responsible for oxidative injury [34]. In this study, the bioassay-guided isolation technique was applied by using in vitro DPPH and ABTS free radical scavenging and XO assays to examine the antioxidant and XOI properties of different polarity extracts and chromatographic fractions. This led to the successful isolation of thirteen components, including six flavonoids (**C1**, **C2**, **C3**, **C4**, **MC5** and **C10**) and seven kavalactones (**MC5**, **C6**, **MC7**, **C8** and **MC9)**. Quantitative analyses revealed various degrees of antioxidant effects in the identified compounds. Among the isolated fractions, flavanones exhibited superior activity as compared with kavalactones (Table 4 and Table 5). The flavonoid **C3**, with a catechol moiety on the B ring, showed the strongest antioxidant activity in both antioxidant assays. This was followed by **C4**, **C2** and **C1**, which are structured with more methoxylated groups. In terms of XO-inhibitory activity, the presence of a free hydroxyl group in the flavonoid **C10** might be associated with a stronger inhibitory effect compared with the compound **C4** (Table 5). The obtained results are consistent with the findings of earlier studies that emphasized the important role of the number and location of OH groups in the radical scavenging and XO activity of flavonoids [35,36,37,38,39,40,41]. Moreover, flavonoids having both XOI and antioxidant activity might be of advantage as potentially applicable compounds to treat gout [34].

Kava lactones have been demonstrated to be involved in the therapeutic value of kava root in the previous literature. In this study, the antioxidant and XOI potential of other constituents besides flavonoids was found in the compound DK (**C6**) and the mixture of DK and 2′,4′-dihydroxy-6′-methoxydihydrochalcone (**MC5**). Among them, **MC5** exerted a synergistic effect to inhibit ABTS free radicals, which resulted in higher radical scavenging activity compared with other kavalactones (**C6**, **MC7**, **C8** and **MC9**) (Supplementary Table S1). However, in comparison with the compound **C6**, the presence of 2′,4′-dihydroxy-6′-methoxydihydrochalcone reduced the antioxidant activity of the mixture **MC5** assayed by the DPPH method. In the present study, yangonin (**MC7**) did not show an antioxidant capacity. This outcome is contrary to that of a previous study that indicated that yangonin exhibited DPPH radical scavenging ability at 64.7 at 2.5 mg/mL. The discrepancy may be due to its kinetic characteristic. Wu et al. [42] stated that longer reaction times resulted in higher scavenging activity for yangonin. In our experiment, 30 min of reaction might not have been sufficient to cause a reaction, which led to the negligible antioxidant capacity of this compound. In the XOI assay, the compound DK (**C6**) was also the most effective inhibitor among the isolated kavalactones.

Herein, we also reported the antibacterial properties of different extracts and isolated constituents of kava root against the growth of five Gram-positive and -negative bacteria, namely *E. coli, K. pneumoniae, L. monocytogenes, B. subtilis* and *P. mirabilis* (Table 6). These bacterial strains are ubiquitous and rapidly growing pathogens that are responsible for life-threatening infections in humans. When employing the agar diffusion method, the EtOAc extract showed antibacterial activity against the *E. coli* strain. However, no tested compound (**MC5**, **C6**, **MC7**, **C8**, **MC9** and **C10**) was found to be involved in the antibacterial activity to constrain this indicator organism. The present result implies that the relatively wide range of antibacterial properties of this extract might be due to the combined modes of active components rather than the individually isolated compounds. Additionally, although the flavanones **C1**–**C4** possess inhibitory effects towards these bacteria, they still require further assessment. Several detected components isolated from the EtOAc extract were revealed to be associated with the antibacterial activity of kava root against other tested pathogens. The fractions **MC5** and **C6** were observed to suppress the growth of *L. monocytogenes*, with the corresponding zones of inhibition of 13 mm and 9 mm. The results indicated that the combination of kavalactone and chalcone (**MC5**) produced a stronger inhibitory effect against a wide spectrum of *L. monocytogenes* as compared to that of single compound DK (**C6**). Although the EtOAc extract had no effects on *K. pneumoniae*, **MC7** displayed slight antibacterial activity towards this bacterium (inhibition zone = 9.67 mm). The mechanism of action of these compounds in inhibiting bacterial strains should be further studied. This study is the first successful investigation of the antibacterial activity of kava root in vitro. The antibacterial properties of the hexane and chloroform extracts were found to inhibit *P. mirabilis* and *B. subtilis*, while those of the other polar extracts were not observed. Elzaawely et al. [30] noted that the growth-inhibitory ability of non-polar extracts (hexane and chloroform) against bacteria might be associated with the presence of several compounds, including oleoresins and sterols. Therefore, the isolation of antibacterial compounds from these extracts should be further investigated.

In this work, we first conducted the isolation, purification and characterization of the components isosakuranetin (**C3)**, 2′,4′-dihydroxy-6′-methoxydihydrochalcone (**MC5**) and alpinetin (**C10**) from kava root. The compound 2′,4′-dihydroxy-6′-methoxydihydrochalcone (**MC5**) was previously identified in *Populus candicans* [43], but its biological properties have not been documented. Compared to earlier reports concerning the antioxidant activity of constituents isolated from kava root, the compound **C3** exhibited the strongest activity. The flavonoid compound alpinetin (**C10**) has been widely found in many different natural sources, such as honey, Thai ginger (*Boesenbergia pandurata*), *Polygonum limbatum* and *Propolis* [44]. The pharmacological investigations of this compound have reported diverse bioactivity, including anticancer, anti-inflammatory, anti-proliferation and antioxidant effects [45]. In contrast, the radical scavenging activity of pinostrobin was not detected in this work, which might be due to the effects of the different methods, as described earlier. However, in this study, the potent anti-hyperuricemia property of pinostrobin was recorded for the first time.

## 5. Conclusions

Kava beverages have a long history and play an integral role in the Pacific Island culture. The findings of this study indicate the additional therapeutic value of this herbal plant. Kava root contains bioactive compounds that possess strong antioxidant activity and potent antibacterial properties. By using elution combining hexane and ethyl acetate at ratios of 9:1 and 8:2, the compounds isosakuranetin (**C3**) and 2′,4′-dihydroxy-6′-methoxydihydrochalcone (**MC5**) were detected and purified for the first time in *P. methysticum* root. Moreover, the results of antioxidant assays revealed that they achieved the highest antioxidant capacity compared to other constituents identified in kava root thus far. The antibacterial activity of various extracts and different compounds of this herbal plant was also successfully assayed in in vitro experiments. Moreover, bioassay-guided fractionation was also established to exploit the potential natural therapeutic agents of this medicinal plant, particularly its antioxidant, anti-hyperuricemia and antibacterial properties.

## Figures and Tables

**Table 1:** Identification of isolated compounds from EtOAc extract of kava root by GC–MS, ESI–MS, ^1^H and ^13^C NMR.

Code	Retention Time	Peak Area (%)	Compound	Chemical Formula	Molecular Weight	Chemical Class
**C1**	23.4	93.28	5-Hydroxy-4′,7-dimethoxyflavanone	C_17_H_16_O_5_	300	Flavonoids
**C2**	25.37	90.00	Matteucinol	C_18_H_18_O_5_	314	Flavonoids
**C3**	21.67	97.17	5,7-Dihydroxy-4′-methoxyflavanone (Isosakuranetin)	C_16_H_14_O_5_	286	Flavonoids
**C4**	23.05	90.00	5,7- Dimethoxyflavanone	C_17_H_16_O_4_	284	Flavonoids
**MC5**	20.84	72.05	5,6-Dehydrokawain (DK)	C_14_H_12_O_3_	228	Kava lactones
	22.26	27.95	2′,4′-Dihydroxy-6′-methoxydihydrochalcone	C_16_H_16_O_4_	272	Flavonoids
**C6**	20.8	94.66	5,6-Dehydrokawain (DK)	C_14_H_12_O_3_	228	Kava lactones
**MC7**	20.07 23.34	59.42 40.58	Kavain Yangonin	C_14_H_14_O_3_ C_15_H_14_O_4_	230 258	Kava lactones Kava lactones
**C8**	23.34	93.64	Yangonin	C_15_H_14_O_4_	258	Kava lactones
**MC9**	16.25 18.35 22.45 23.4	12.78 20.44 54.77 8.9	Dihydro-5,6-dehydrokavain (DDK) 7,8-Dihydrokavain Dihydromethysticin Methysticin	C_14_H_14_O_3_ C_14_H_16_O_3_ C_15_H_16_O_5_ C_15_H_14_O_5_	230 232 276 274	Kava lactones Kava lactones Kava lactones Kava lactones
**C10**	21.61	90.06	Alpinetin	C_16_H_14_O_4_	270	Flavonoids

**Table 2:** Xanthine oxidase inhibitory and antioxidant activity of kava root extracts as IC_50_ values.

Extracts	Radical Scavenging Activity		XOI Activity
	IC_50_ DPPH (µg/mL)	IC_50_ ABTS (µg/mL)	IC_50_ (µg/mL)
Hexane	-	1706.0 ± 4.6 a	2008.1 ± 6.7 a
Chloroform	2392.3 ± 4.5 a	1304.0 ± 5.3 b	922.7 ± 3.1 b
Ethyl acetate	946.3 ± 7.6 b	1007.0 ± 6.2 c	724.6 ± 2.4 b
Aqueous	971.6 ± 9.0 b	1017.4 ± 4.7 c	-

Data are presented as means ± SD (standard deviation). Mean values with different lowercase letters indicate significant differences in the same column (*p* < 0.05) (*n* = 3).

**Table 3:** Antibacterial activity of the kava root extracts.

	Zone of Inhibition (mm)				
	*E. coli* ^(a)^	*K. pneumoniae* ^(a)^	*P. mirabilis* ^(a)^	*B. subtilis* ^(b)^	*L. monocytogenes* ^(b)^
Hexane	-	-	10.1 ± 2.5	-	-
Chloroform	-	-	-	15.0 ± 2.0	-
EtOAc	23.0 ± 2.5	-	-	-	-
Water	-	-	-	-	-
Control	-	-	-	-	-
Standards (30 µg/disc)					
Ampicillin	34.0 ± 1.0	44.1 ± 3.2	43.0 ± 1.5	17.1 ± 1.5	24.0 ± 3.1
Streptomycin	19.1 ± 1.0	15.2 ± 0.6	29. ± 1.5	12.0 ± 1.0	18.1 ± 1.7

-: no inhibition, data are presented as means ± SD (standard deviation) (*n* = 3). ^(a)^: Gram-negative bacteria; ^(b)^: Gram-positive bacteria.

**Table 4:** Antioxidant activity of compounds isolated from kava root.

Compounds	IC_50_ (µg/mL)	
	DPPH	ABTS
**C1**	-	205.8 ± 5.7 c
**C2**	-	172.8 ± 6.5 d
**C3**	74.8 ± 3.5 e	76.5 ± 7.1 f
**C4**	477.8 ± 4.7 d	143.5 ± 6.5 e
**MC5**	1211.5 ± 6.1 a	183.4 ± 5.7 d
**C6**	496.6 ± 4.5 c	463.5 ± 7.1 b
**BHT**	9.5 ± 0.2 f	45.1 ± 3.7 g

Data are presented as means ± SD (standard deviation). Mean values with different lowercase letters indicate significant differences in the same column (*p* < 0.05) (*n* = 3). -: no inhibition.

**Table 5:** Xanthine oxidase inhibitory activity of fractions isolated from kava root.

Fractions	IC_50_ (µg/mL)
**C4**	268.65 ± 2.00 c
**MC5**	452.68 ± 3.43 b
**C6**	242.01 ± 1.12 d
**MC9**	465.27 ± 2.22 a
**C10**	134.52 ± 1.80 e
Allopurinol *	21.33 ± 0.19 f

Data are presented as means ± SD (standard deviation). Mean values with different lowercase letters indicate significant differences in the same column (*p* < 0.05) (*n* = 3). *: positive control.

**Table 6:** Antibacterial activity of the isolated compounds extracted from kava root.

	Zone of Inhibition (mm)				
	*E. coli* ^(a)^	*K. pneumoniae* ^(a)^	*P. mirabilis* ^(a)^	*B. subtilis* ^(b)^	*L. monocytogenes* ^(b)^
**MC5**	-	-	-	-	13.0 ± 0.5
**C6**	-	-	-	-	9.0 ± 0.5
**MC7**	-	9.7 ± 0.5	-	-	-
Control	-	-	-	-	-
Standards (30 µg/disc)					
Ampicillin	34.0 ± 1.0	44.1 ± 3.2	43.0 ± 1.5	17.1 ± 1.5	24.0 ± 3.1
Streptomycin	19.1 ± 1.0	15.2 ± 0.6	29. ± 1.5	12.0 ± 1.0	18.1 ± 1.7

Data are presented as means ± SD (standard deviation). -: no inhibition. ^(a)^: Gram-negative bacteria; ^(b)^: Gram-positive bacteria.

## Data Availability

The data presented in this study are available on request from the corresponding author.

## Supplementary Materials

The following supporting information can be downloaded at: https://www.mdpi.com/article/10.3390/foods11233889/s1, Figure S1 (GC-MS chromatography of **C3**), Figure S2 (ESI-MS spectrum of **C3**), Figure S3.1 (1H spectrum of **C3**), Figure S3.2 (13C spectrum of **C3**), Figure S4.1 (LC-MS of **C3**), Figure S4.2 (ESI-MS of **C3**), Figure S5 (GC-MS chromatography of **C4**), Figure S6 (ESI-MS spectrum of **C4**), Figure S7 (GC-MS chromatography of **C5**), Figure S8 (ESI-MS spectrum of **C5**), Figure S9 (GC-MS chromatography of **C10**), Figure S10 (ESI-MS spectrum of **C10**) and Table S1 (X-ray and NMR results of identified compounds from kava root) were provided.
