# Supplementary material for: Isolation and Identification of Constituents Exhibiting Antioxidant, Antibacterial, and Antihyperuricemia Activities in Piper methysticum Root
Source: Foods. 2022 Dec 1;11(23):3889. doi: 10.3390/foods11233889 (PMC9739624; doi:10.3390/foods11233889)

checkCIF/PLATON report

Structure factors have been supplied for datablock(s) I

THIS REPORT IS FOR GUIDANCE ONLY. IF USED AS PART OF A REVIEW PROCEDURE FOR PUBLICATION, IT SHOULD NOT REPLACE THE EXPERTISE OF AN EXPERIENCED CRYSTALLOGRAPHIC REFEREE.

No syntax errors found. CIF dictionary Interpreting this report

Datablock: I

```
Bond precision:  C-C = 0.0033 A          Wavelength=0.71073

Cell:            a=5.1478(10)     b=9.2061(18)       c=28.997(6)
                 alpha=90         beta=92.721(3)     gamma=90
Temperature:     173 K

                Calculated                 Reported
Volume          1372.7(5)                  1372.7(5)
Space group     P 21/c                     P 1 21/c 1
Hall group      -P 2ybc                    -P 2ybc
Moiety formula  C16 H14 O4, O              ?
Sum formula     C16 H14 O5                 C16 H14 O5
Mr              286.27                     286.27
Dx,g cm-3       1.385                      1.385
Z               4                          4
Mu (mm-1)       0.104                      0.104
F000            600.0                      600.0
F000'           600.36
h,k,lmax        6,12,38                    6,12,38
Nref            3301                       3297
Tmin,Tmax       0.994,0.998                0.880,1.000
Tmin'           0.968

Correction method= # Reported T Limits: Tmin=0.880 Tmax=1.000
AbsCorr = MULTI-SCAN

Data completeness= 0.999          Theta(max)= 27.980

R(reflections)= 0.0560( 2203)     wR2(reflections)= 0.1528( 3297)

S = 1.035                  Npar= 192
```

The following ALERTS were generated. Each ALERT has the format
test-name_ALERT_alert-type_alert-level.
Click on the hyperlinks for more details of the test.

Alert level B

```
PLAT306_ALERT_2_B Isolated Oxygen Atom (H-atoms Missing ?) .......         O6 Check
PLAT430_ALERT_2_B Short Inter D...A Contact  O2       ..O6       .       2.80 Ang.
                                                  x,1+y,z  =      1_565 Check
```

Alert level C

```
DIFMX02_ALERT_1_C  The maximum difference density is > 0.1*ZMAX*0.75
            The relevant atom site should be identified.
PLAT048_ALERT_1_C MoietyFormula Not Given (or Incomplete) ........     Please Check
PLAT094_ALERT_2_C Ratio of Maximum / Minimum Residual Density ....       2.71 Report
PLAT097_ALERT_2_C Large Reported Max.  (Positive) Residual Density       0.69 eA-3
PLAT975_ALERT_2_C Check Calcd Resid. Dens.  1.04A   From O6              0.56 eA-3
PLAT975_ALERT_2_C Check Calcd Resid. Dens.  1.02A   From O6              0.45 eA-3
```

Alert level G

```
PLAT007_ALERT_5_G Number of Unrefined Donor-H Atoms ..............          1 Report
PLAT793_ALERT_4_G Model has Chirality at C7          (Centro SPGR)          R Verify
PLAT912_ALERT_4_G Missing # of FCF Reflections Above STh/L=  0.600          5 Note
PLAT978_ALERT_2_G Number C-C Bonds with Positive Residual Density.          6 Info
```

0 **ALERT level A** = Most likely a serious problem - resolve or explain
2 **ALERT level B** = A potentially serious problem, consider carefully
6 **ALERT level C** = Check. Ensure it is not caused by an omission or oversight
4 **ALERT level G** = General information/check it is not something unexpected

2 ALERT type 1 CIF construction/syntax error, inconsistent or missing data
7 ALERT type 2 Indicator that the structure model may be wrong or deficient
0 ALERT type 3 Indicator that the structure quality may be low
2 ALERT type 4 Improvement, methodology, query or suggestion
1 ALERT type 5 Informative message, check

checkCIF publication errors

Alert level A

```
PUBL003_ALERT_1_A  The contact author's name is missing,
            _publ_contact_author_name.
PUBL005_ALERT_1_A  _publ_contact_author_email, _publ_contact_author_fax and
            _publ_contact_author_phone are all missing.
            At least one of these should be present.
PUBL006_ALERT_1_A  _publ_requested_journal is missing
            e.g. 'Acta Crystallographica Section C'
PUBL009_ALERT_1_A  _publ_author_name is missing. List of author(s) name(s).
PUBL010_ALERT_1_A  _publ_author_address is missing. Author(s) address(es).
```

5 **ALERT level A** = Data missing that is essential or data in wrong format
0 **ALERT level G** = General alerts. Data that may be required is missing

Publication of your CIF

You should attempt to resolve as many as possible of the alerts in all categories. Often the minor alerts point to easily fixed oversights, errors and omissions in your CIF or refinement strategy, so attention to these fine details can be worthwhile. In order to resolve some of the more serious problems it may be necessary to carry out additional measurements or structure refinements. However, the nature of your study may justify the reported deviations from journal submission requirements and the more serious of these should be commented upon in the discussion or experimental section of a paper or in the "special_details" fields of the CIF. *checkCIF* was carefully designed to identify outliers and unusual parameters, but every test has its limitations and alerts that are not important in a particular case may appear. Conversely, the absence of alerts does not guarantee there are no aspects of the results needing attention. It is up to the individual to critically assess their own results and, if necessary, seek expert advice.

If level A alerts remain, which you believe to be justified deviations, and you intend to submit this CIF for publication in a journal, you should additionally insert an explanation in your CIF using the Validation Reply Form (VRF) below. This will allow your explanation to be considered as part of the review process.

Validation response form

Please find below a validation response form (VRF) that can be filled in and pasted into your CIF.

```
# start Validation Reply Form
_vrf_PUBL003_GLOBAL
;
PROBLEM: The contact author’s name is missing,
RESPONSE: ...
;
_vrf_PUBL005_GLOBAL
;
PROBLEM: _publ_contact_author_email, _publ_contact_author_fax and
RESPONSE: ...
;
_vrf_PUBL006_GLOBAL
;
PROBLEM: _publ_requested_journal is missing
RESPONSE: ...
;
_vrf_PUBL009_GLOBAL
;
PROBLEM: _publ_author_name is missing. List of author(s) name(s).
RESPONSE: ...
;
_vrf_PUBL010_GLOBAL
;
PROBLEM: _publ_author_address is missing. Author(s) address(es).
RESPONSE: ...
;
# end Validation Reply Form
```

If you wish to submit your CIF for publication in Acta Crystallographica Section C or E, you should upload your CIF via the web. If you wish to submit your CIF for publication in IUCrData you should upload your CIF via the web. If your CIF is to form part of a submission to another IUCr journal, you will be asked, either during electronic submission or by the Co-editor handling your paper, to upload your CIF via our web site.

PLATON version of 19/10/2018; check.def file version of 15/10/2018

Datablock I - ellipsoid plot

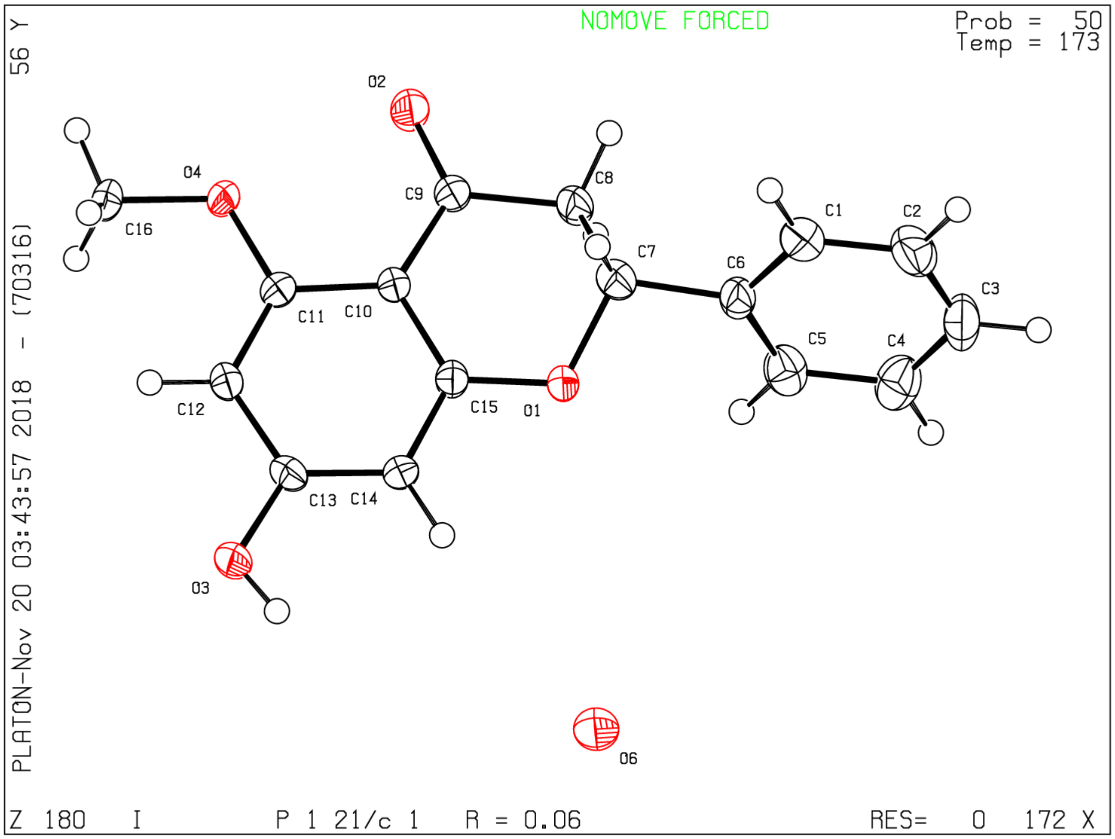

Supplement: Supplementary file 1 [file foods-11-03889-s001.zip › foods-1980133-supplementary/Supplementary data/CIF2. compound C10.pdf]
