# Supplementary material for: Isolation and Identification of Constituents Exhibiting Antioxidant, Antibacterial, and Antihyperuricemia Activities in Piper methysticum Root
Source: Foods. 2022 Dec 1;11(23):3889. doi: 10.3390/foods11233889 (PMC9739624; doi:10.3390/foods11233889)
Supplement: Supplementary file 1 [file foods-11-03889-s001.zip › foods-1980133-supplementary/Supplementary data/Figure S2. ESI-MS spectrum of compound 3.pdf]

EIS-MS 171196MS_Xuan.pdf - Adobe Acrobat Reader DC
ファイル 編集 表示(V) ウィンドウ(W) ヘルプ(H)
ホーム ツール EIS-MS 171196MS_X...
17 / 21 101%
サインイン

C:\Xcalibur\...\170926A\170926_03 2017/09/26 14:03:33 (2) V4
MeOH soln.

170926_03 #7 RT: 0.07 AV: 1 NL: 1.27E6
T: FTMS + p ESI Full ms [100.00-2000.00]

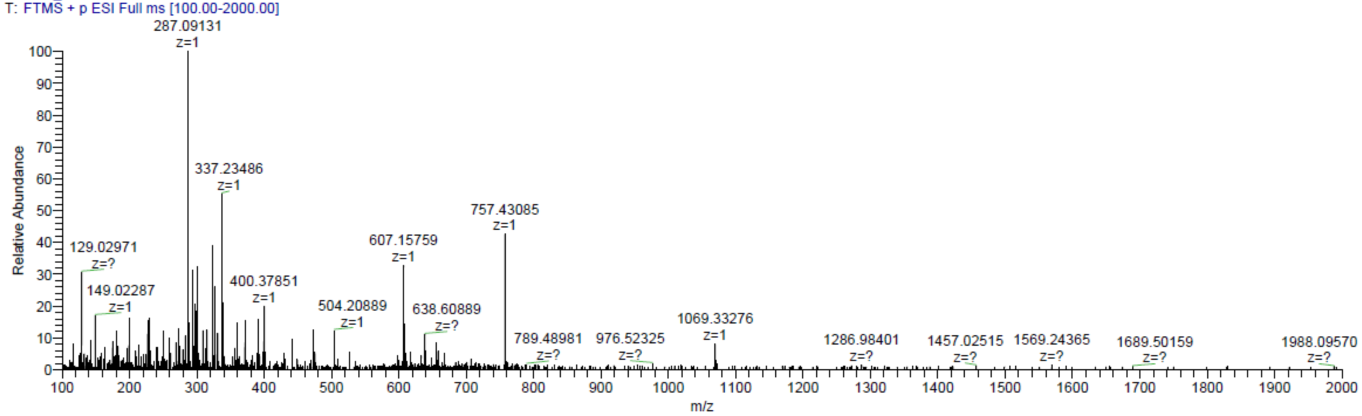

170926_03 #7 RT: 0.07 AV: 1 NL: 1.27E6
T: FTMS + p ESI Full ms [100.00-2000.00]

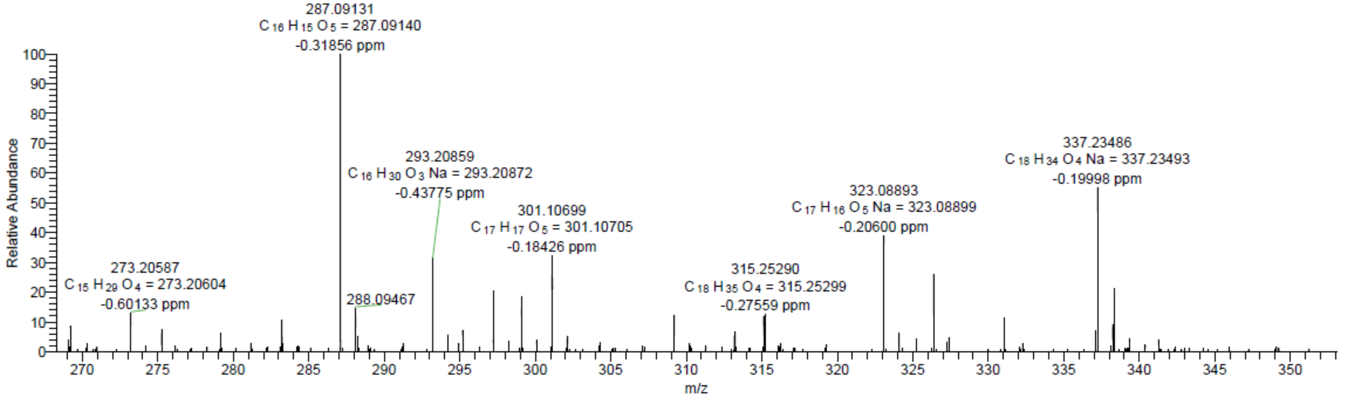

PDF を書き出し
Adobe Export PDF
オンラインで PDF ファイルを Word または Excel に変換します
PDF ファイルを選択
EIS-MS 171196MS_Xuan.pdf
次に変換：
Microsoft Word (*.docx)
文書の言語：
日本語 変更
変換
PDF を作成
PDF を編集
注釈
ファイルを結合
入力と署名
署名用に送信
送信とトラック
Acrobat Pro DCで PDFを変換・編集
今すぐ無料でお試し
16:45 2018/09/09
