# Supplementary material for: Isolation and Identification of Constituents Exhibiting Antioxidant, Antibacterial, and Antihyperuricemia Activities in Piper methysticum Root
Source: Foods. 2022 Dec 1;11(23):3889. doi: 10.3390/foods11233889 (PMC9739624; doi:10.3390/foods11233889)
Supplement: Supplementary file 1 [file foods-11-03889-s001.zip › foods-1980133-supplementary/Supplementary data/Figure S3.1. 1H spectrum of compound 3.pdf]

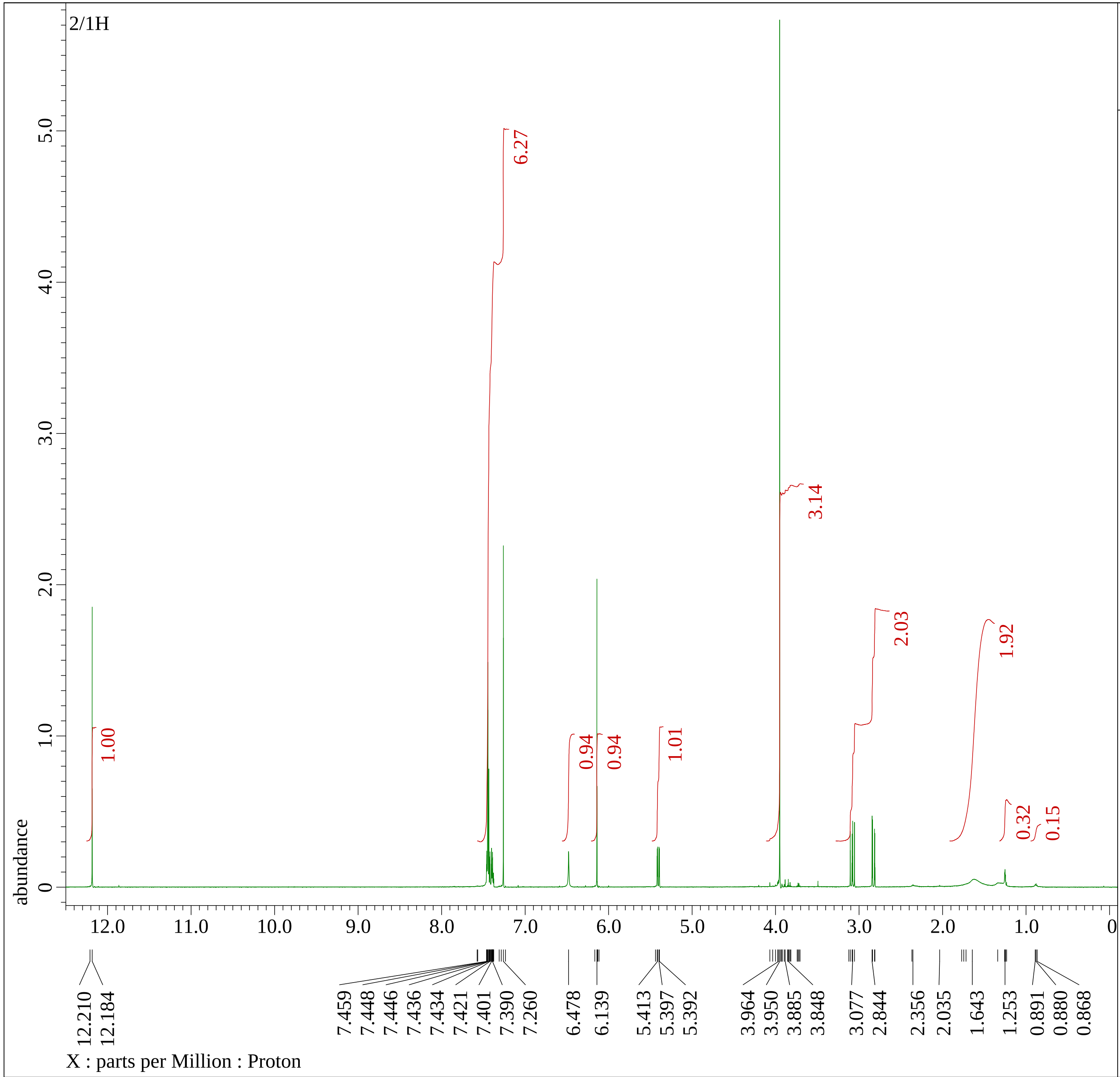

Filename = Van_2_1H-1-4.jdf
Author = delta
Experiment = proton.jxp
Sample_Id = Van_170925_2
Solvent = CHLOROFORM-D
Creation_Time = 25-SEP-2017 14:02:29
Revision_Time = 26-SEP-2017 15:45:34
Current_Time = 26-SEP-2017 15:46:41

Comment = 2/1H
Data_Format = 1D COMPLEX
Dim_Size = 26214
Dim_Title = Proton
Dim_Units = [ppm]
Dimensions = X
Spectrometer = DELTA2_NMR

Field_Strength = 14.09636928[T] (600[MHz]
X_Acq_Duration = 2.9097984[s]
X_Domain = 1H
X_Freq = 600.1723046[MHz]
X_Offset = 5[ppm]
X_Points = 32768
X_Prescans = 1
X_Resolution = 0.34366642[Hz]
X_Sweep = 11.26126126[kHz]
X_Sweep_Clipped = 9.00900901[kHz]
Irr_Domain = Proton
Irr_Freq = 600.1723046[MHz]
Irr_Offset = 5[ppm]
Tri_Domain = Proton
Tri_Freq = 600.1723046[MHz]
Tri_Offset = 5[ppm]
Clipped = FALSE
Scans = 32
Total_Scans = 32

Relaxation_Delay = 5[s]
Recvr_Gain = 52
Temp_Get = 24[dC]
X_90_Width = 13.5[us]
X_Acq_Time = 2.9097984[s]
X_Angle = 45[deg]
X_Atn = 9.8[dB]
X_Pulse = 6.75[us]
Irr_Mode = Off
Tri_Mode = Off
Dante_Presat = FALSE
Initial_Wait = 1[s]
Repetition_Time = 7.9097984[s]
