# Supplementary material for: Isolation and Identification of Constituents Exhibiting Antioxidant, Antibacterial, and Antihyperuricemia Activities in Piper methysticum Root
Source: Foods. 2022 Dec 1;11(23):3889. doi: 10.3390/foods11233889 (PMC9739624; doi:10.3390/foods11233889)
Supplement: Supplementary file 1 [file foods-11-03889-s001.zip › foods-1980133-supplementary/Supplementary data/Figure S3.2. 13C spectrum of compound 3.pdf]

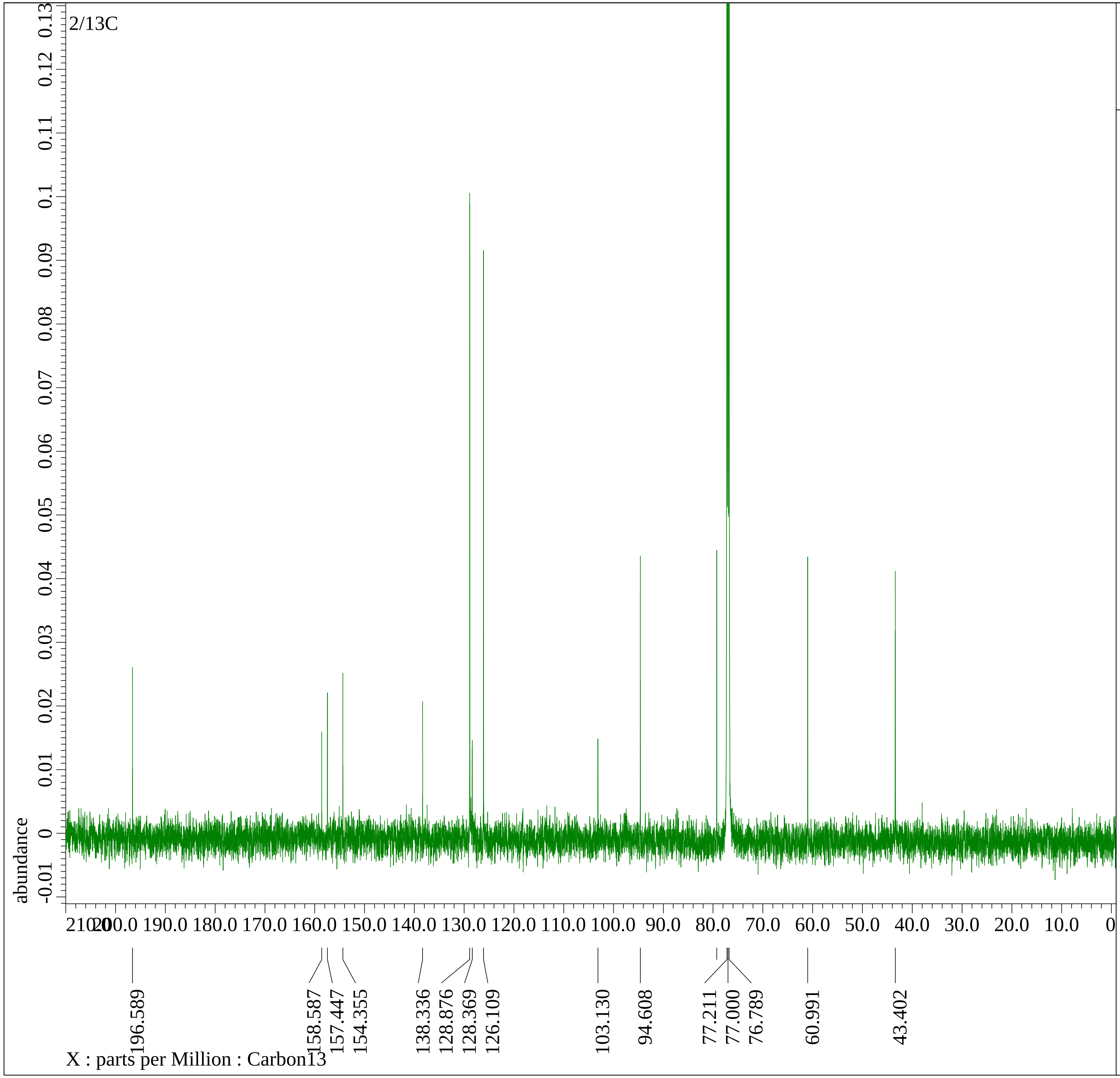

JEOL
Solutions for Innovation

Filename	=	Van_2_13C-1-3.jdf
Author	=	delta
Experiment	=	carbon.jxp
Sample_Id	=	Van_170925_2
Solvent	=	CHLOROFORM-D
Creation_Time	=	25-SEP-2017 14:07:15
Revision_Time	=	26-SEP-2017 15:49:34
Current_Time	=	26-SEP-2017 15:50:11
Comment	=	2/13C
Data_Format	=	1D COMPLEX
Dim_Size	=	26214
Dim_Title	=	Carbon13
Dim_Units	=	[ppm]
Dimensions	=	X
Spectrometer	=	DELTA2_NMR
Field_Strength	=	14.09636928[T] (600[MHz]
X_Acq_Duration	=	0.69206016[s]
X_Domain	=	13C
X_Freq	=	150.91343039[MHz]
X_Offset	=	100[ppm]
X_Points	=	32768
X_Prescans	=	4
X_Resolution	=	1.44496109[Hz]
X_Sweep	=	47.34848485[kHz]
X_Sweep_Clipped	=	37.87878788[kHz]
Irr_Domain	=	Proton
Irr_Freq	=	600.1723046[MHz]
Irr_Offset	=	5[ppm]
Clipped	=	TRUE
Scans	=	4000
Total_Scans	=	4000
Relaxation_Delay	=	2[s]
Recvr_Gain	=	60
Temp_Get	=	24.6[dC]
X_90_Width	=	10.2[us]
X_Acq_Time	=	0.69206016[s]
X_Angle	=	30[deg]
X_Atn	=	11.1[dB]
X_Pulse	=	3.4[us]
Irr_Atn_Dec	=	24.81[dB]
Irr_Atn_Noe	=	24.81[dB]
Irr_Noise	=	WALTZ
Irr_Pwidth	=	76[us]
Decoupling	=	TRUE
Initial_Wait	=	1[s]
Noe	=	TRUE
Noe_Time	=	2[s]
Repetition_Time	=	2.69206016[s]
