# Supplementary material for: Isolation and Identification of Constituents Exhibiting Antioxidant, Antibacterial, and Antihyperuricemia Activities in Piper methysticum Root
Source: Foods. 2022 Dec 1;11(23):3889. doi: 10.3390/foods11233889 (PMC9739624; doi:10.3390/foods11233889)

ESI-MS spectrum (Positive ion, enlarged)

HIROSHIMA UNIVERSITY

No. 4

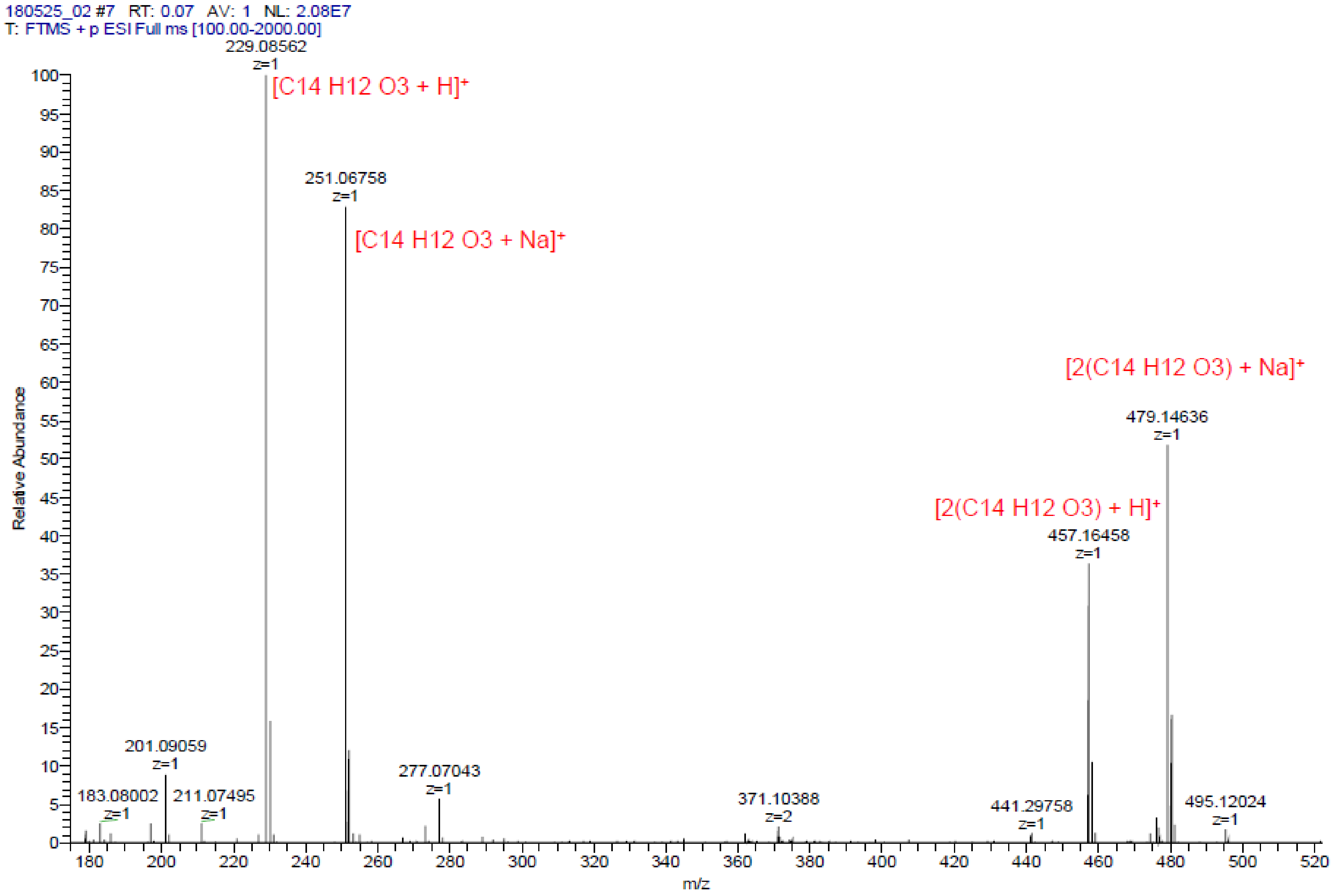

ESI-MS spectrum (Negative ion mode)

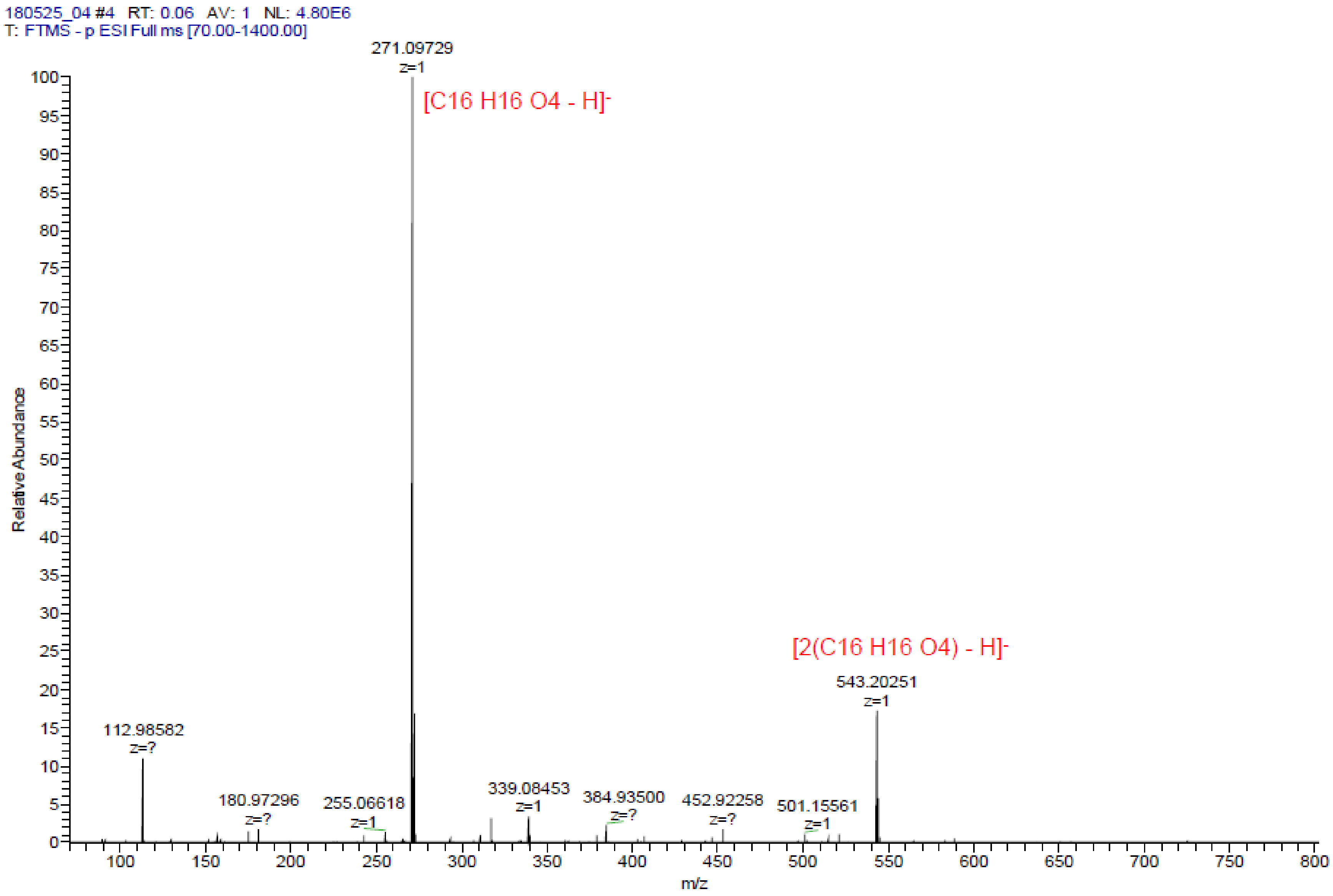

Supplement: Supplementary file 1 [file foods-11-03889-s001.zip › foods-1980133-supplementary/Supplementary data/Figure S8. ESI-MS spectrum of compound 5.pdf]
