# Supplementary material for: Isolation and Identification of Constituents Exhibiting Antioxidant, Antibacterial, and Antihyperuricemia Activities in Piper methysticum Root
Source: Foods. 2022 Dec 1;11(23):3889. doi: 10.3390/foods11233889 (PMC9739624; doi:10.3390/foods11233889)
Supplement: Supplementary file 1 [file foods-11-03889-s001.zip › foods-1980133-supplementary/Supplementary data/Figure S9. GC-MS chromatography of compound 10.pdf]

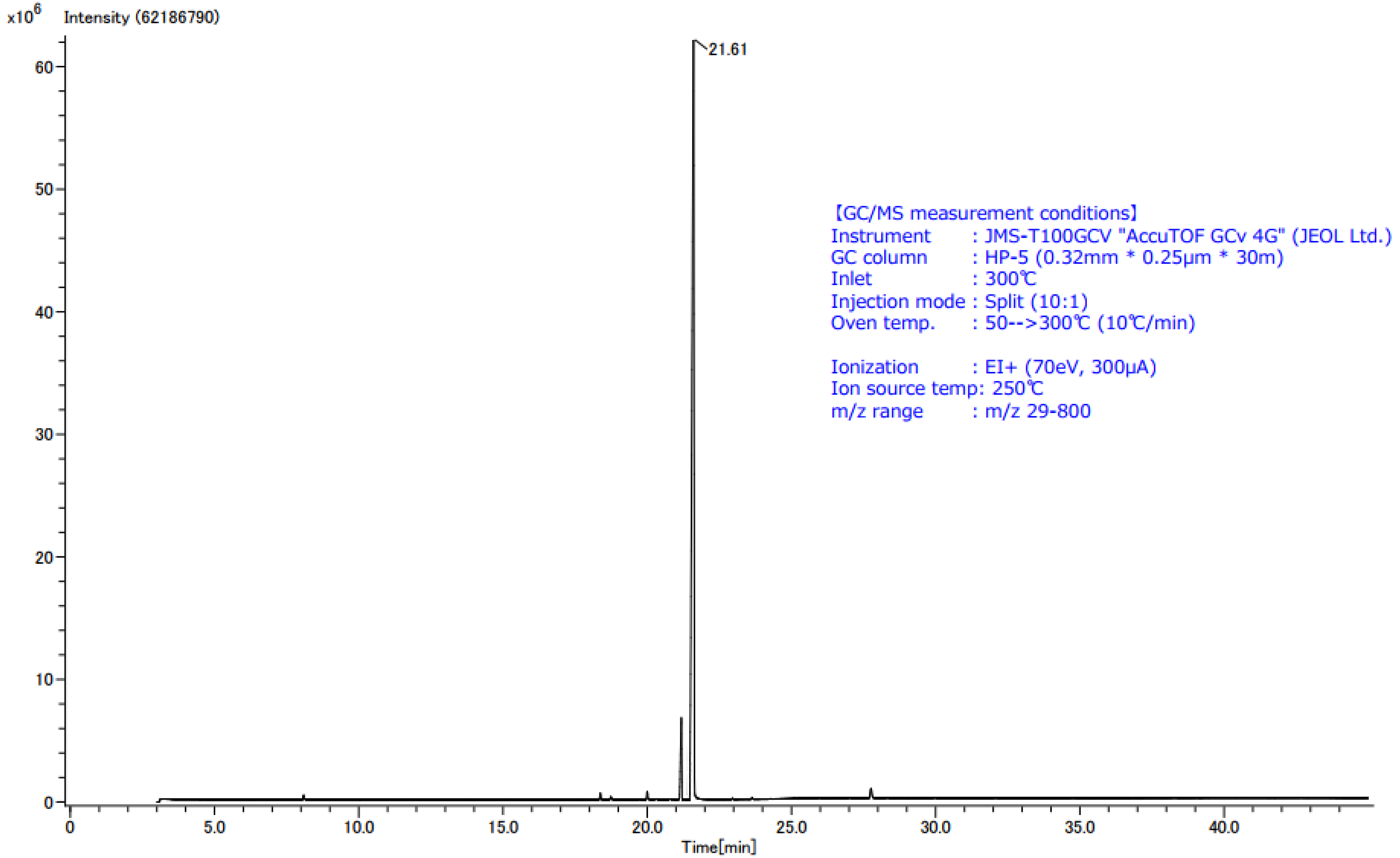
x10^6 Intensity (62186790)
21.61
【GC/MS measurement conditions】
Instrument : JMS-T100GCV "AccuTOF GCv 4G" (JEOL Ltd.)
GC column : HP-5 (0.32mm * 0.25μm * 30m)
Inlet : 300℃
Injection mode : Split (10:1)
Oven temp. : 50-->300℃ (10℃/min)
Ionization : EI+ (70eV, 300μA)
Ion source temp: 250℃
m/z range : m/z 29-800
0
10
20
30
40
50
60
0
5.0
10.0
15.0
20.0
25.0
30.0
35.0
40.0
Time[min]
