# Supplementary material for: Isolation and Identification of Constituents Exhibiting Antioxidant, Antibacterial, and Antihyperuricemia Activities in Piper methysticum Root
Source: Foods. 2022 Dec 1;11(23):3889. doi: 10.3390/foods11233889 (PMC9739624; doi:10.3390/foods11233889)
Supplement: Supplementary file 1 [file foods-11-03889-s001.zip › foods-1980133-supplementary/Supplementary data/Supplementary Table S1.pdf]

Supplementary Table S1. X-ray and NMR results of identified compounds from kava root

5-hydroxy-4′,7-dimethoxyflavanone (**C1**). Compound **C1** was isolated as a white powder from the combined fractions 14–17, which were eluted with Hexane:EtOAc 9:1. This compound was purified after eluting by hexane and filtering through a filter paper to remove contaminants. The identification as 5-hydroxy-4′,7-dimethoxyflavanone (Figure 2) was obtained by analysis of its ^{1}H- and ^{13}C-NMR spectra and literature data [26]. 1H-NMR δ (ppm): 12.8 (1H, s); 7.78(2H, dd, 8 Hz, 4 Hz, H2′ and H6′); 6.97 (2H, dd, 8 Hz, 4 Hz, H3′and H5′); 6.52 (1H, s, H3); 6.43 (1H, d,4 Hz, H8); 6.32 (1H, d, 4 Hz, H6); 3.86(3H, s); 3.85 (3H, s).13C-NMR δ (ppm): 163.9 (C2), 104.2 (C3),182.4 (C4), 162.0 (C5), 98.0 (C6), 165.3 (C7), 92.5 (C8), 157.6 (C9), 105.4 (C10), 123.4 (C1′), 127.9 (C2′), 114.4 (C3′), 162.5 (C4′), 114.4(C5′), 127.9 (C6′), 55.8 (C6′–OCH3), 55.5(C7–OCH3)

Matteucinol (**C2**). Compound **C2** was precipitated as a yellow powder from fractions 20–26 which were eluted with Hexane:EtOAc 9:1. The identification as 5-hydroxy-4′,7-dimethoxyflavanone (Figure 2) was obtained by analysis of its ^{1}H- and ^{13}C-NMR spectra and literature data [26]. ^{1}H-NMR δ (ppm): 2.03(3H, s, 6-CH3), 2.05 (3H, s, 8-CH3), 2.78 (1H, dd, J = 17 Hz, 3 Hz, H-3β), 3.03 (1H, dd, J = 17 Hz,13 Hz, H-3α), 3.83 (3H, s, OCH3), 5.32 (1H, dd, J = 13 Hz, 3 Hz, H-2), 6.95 (2H, d, J = 9 Hz, H-3′,5′), 7.39(2H, d, J = 9 Hz, H-2′,6′), 12.29 (1H, s, chelated 5-OH). ^{13}C-NMR δ (ppm): 6.9 (6-CH3), 7.6 (8-CH3), 43.1(3α, 3β), 55.2 (OCH3), 78.2 (C-1), 102.3 (C-8), 102.7 (C-10), 103.5 (C-6), 114.0 (C-3′,5′), 127.4 (C-2′,6′),131.0 (C-1′), 157.7 (C-9), 158.8 (C-5), 159.6 (C-4′), 162.1 (C-7), 196.5 (C-4)

5,7-dihydroxy-4′-methoxyflavanone (**C3**). Compound **C3** was isolated as a off white powder from the combined fractions 32–40 with Hexane:EtOAc 9:1. Hexane was also used to purify this compound with the purity being evaluated as 97.17% by GC-MS (Supplementary Material, Figure S1). According to ESI-MS data (m/z: 287.09 [M + H]+, Supplementary Material, Figure S2), the molecular formula of compound **C3** was determined as $C_{16}H_{14}O_5$. The ^{1}H- result was interpreted to match 5,7-dihydroxy-4′-methoxyflavanone (Figure 1) by compared with spectroscopic data in the literature [27]. ^{1}H-NMR (400 MHz, CDCI3) 5:2.77 (1H, dd, J = 17.1, 3.0 Hz, H-3a), 3.09 (1H, dd, J= 17.1, 13.0 Hz, H-3b), 3.81 (3H, s, 4'-OMe), 5.33 (1H, dd, J = 13.0, 3.0 Hz, H-2), 5.84 (1H, br s, 7-OH), 5.95 (1H, d, J = 2.2 Hz, H-6), 5.97 (1H, d, J = 2.2 Hz, H-8), 6.93 (2xlH, d, J = 8.7 Hz, H-3', H-5'), 7.35 (2xlH, d, J = 8.7 Hz, H-2', H-6')

5,7- dimethoxyflavanone (**C4**). Compound **C4** was isolated as a off white powder from the combined fractions 4–6 with Hexane:EtOAc 8:2. Hexane was also used to purify this compound with the purity being evaluated as 90% by GC-MS. The molecular formula of compound **C4** was determined as $C_{17}H_{16}O_4$. The identification as 5-hydroxy-4′,7-dimethoxyflavanone (Figure 2) was obtained by literature data [28]. ; ^{1}H NMR (CDCl3) δ 7.37-7.45 (5H, m, H-2', H3', H-4', H-5' and H-6'), 6.15 (1H, d, J = 2.2 Hz, H-8), 6.08 (1H, d, *J* = 2.2 Hz, H-6), 5.40 (1H, dd, *J* = 13.1, 2.9 Hz, H-2), 3.88 (3H, s, OMe-5), 3.81 (3H, s, OMe-7), 3.00 (1H, dd, *J* = 16.5, 13.1 Hz, H-3a), 2.79 (1H, dd, J= 16.5, 2.9 Hz, H-3b); ^{13}C NMR (CDCl3) δ 189.2 (C-4), 166.0 (C-7), 165.0 (C-5), 162.3 (C-8a), 138.7 (C-1'), 128.8 (C-3' and C-5'), 128.6 (C-4'), 126.1 (C-2' and C6'), 106.0 (C-4a), 93.5 (C-8), 93.2 (C-6), 79.2 (C-2), 56.1 (OMe-5), 55.6 (OMe-7), 45.6 (C-3)

5,6-dehydrokawain (**C6**). Compound **C6** was isolated as pale-yellow needles from fractions 11–15 which were eluted with Hexane:EtOAc 8:2. Hexane was also used to purify this compound with

the purity being evaluated as 94.66% by GC-MS. The molecular formula of compound **C6** was determined as $C_{14}H_{12}O_3$. The identification as 5,6-dehydrokawain (Figure 2) was obtained by literature data [29]. ^{1}H NMR (400 MHz): δ 3.83 (s, 3H), 5.50 (d, *J* = 2.0 Hz, 1H), 5.95 (d, *J* = 2.4 Hz, 1H), 6.59 (d, *J* = 16.0 Hz, 1H), 7.35–7.52 (m, 6H); ^{13}C NMR (400 MHz): δ 55.9, 88.8, 101.3, 118.6, 127.4, 128.9, 129.4, 135.2, 135.8, 158.6, 164.0, 171.1

Yangonin (**C8**). Compound **C8** was isolated as light yellow needles from fractions 48–55 which were eluted with Hexane:EtOAc 8:2. Its molecular formula was established to be $C_{15}H_{14}O_4$ on the basis of ESI-MS: m/z 259.1 [M − H]+ (Supplementary Material, Figure S8). The purity of yangonin reached 93.64% according to GC-MS result (Supplementary Material, Figure S7). The compoundwas identified as yangonin (Figure 2) in the literature [30]. ^{1}H-NMR δ (ppm): 5.45 (d, *J* = 2.1 Hz, H-3); 5.87(d, *J* = 2.1 Hz, H-5); 6.43 (d, *J* = 16.0 Hz, H-7); 7.43 (d, *J* = 16.0 Hz, H-8); 7.42 (d, *J* = 8.8 Hz, H-10 andH-14); 6.88 (d, *J* = 8.8 Hz, H-11 and H-13); 3.82 (s, 4-OMe); 3.79 (s, 12-OMe). ^{13}C-NMR δ (ppm): 164.1(C-2), 88.3 (C-3), 171.2 (C-4), 100.4 (C-5), 159.0 (C-6), 116.3 (C-7), 135.4 (C-8), 127.9 (C-9), 1289 (C-10),114.3 (C-11), 160.7 (C-12), 114.3 (C-13), 128.9 (C-14), 55.8 (4-OMe), 55.3 (12-OMe)

Pinostrobin (**C10**). Compound **C10** was isolated as light yellow needles from fractions 8–15 which were eluted with Hexane:EtOAc 7:3. Its molecular formula was established to be $C_{16}H_{14}O_4$ on the basis of ESI-MS: m/z 269.1 [M − H]+ (Supplementary Material, Figure S10). The purity of pinostrobin reached 90.06% according to GC-MS result (Supplementary Material, Figure S9). The compoundwas identified as pinostrobin (Figure 2) in the literature [31]. ^{1}H NMR (400 MHz; CDCl3): δ 2.81 (3- cis), 3.86 (C-7-OMe), 3.10 (3-trans), 5.43 (2H, t, J=6.7), 6.03 (H-8), 6.04 (H-6), 7.28-7.49 (H-2′-H6′), 12.07 (C-5- OH). ^{13}C NMR (100 MHz; CDCl3): δ 43.36 (C-3), 55.60 (C-7-OMe), 79.25 (C-2), 95.49 (C-6, C-8), 103.20 (C-4a), 126.17 (C-2′, C-6′), 128.92 (C-3′, C-4′, C-5′), 138.30 (C-1′), 163.16 (C-5), 164.36 (C-7), 164.63 (C-8a), 195.79 (C-4).
