# Supplementary figures and images for: Isolation and Identification of Constituents Exhibiting Antioxidant, Antibacterial, and Antihyperuricemia Activities in Piper methysticum Root
Source: Foods. 2022 Dec 1;11(23):3889. doi: 10.3390/foods11233889 (PMC9739624; doi:10.3390/foods11233889)

x10<sup>6</sup> Intensity (89761430)

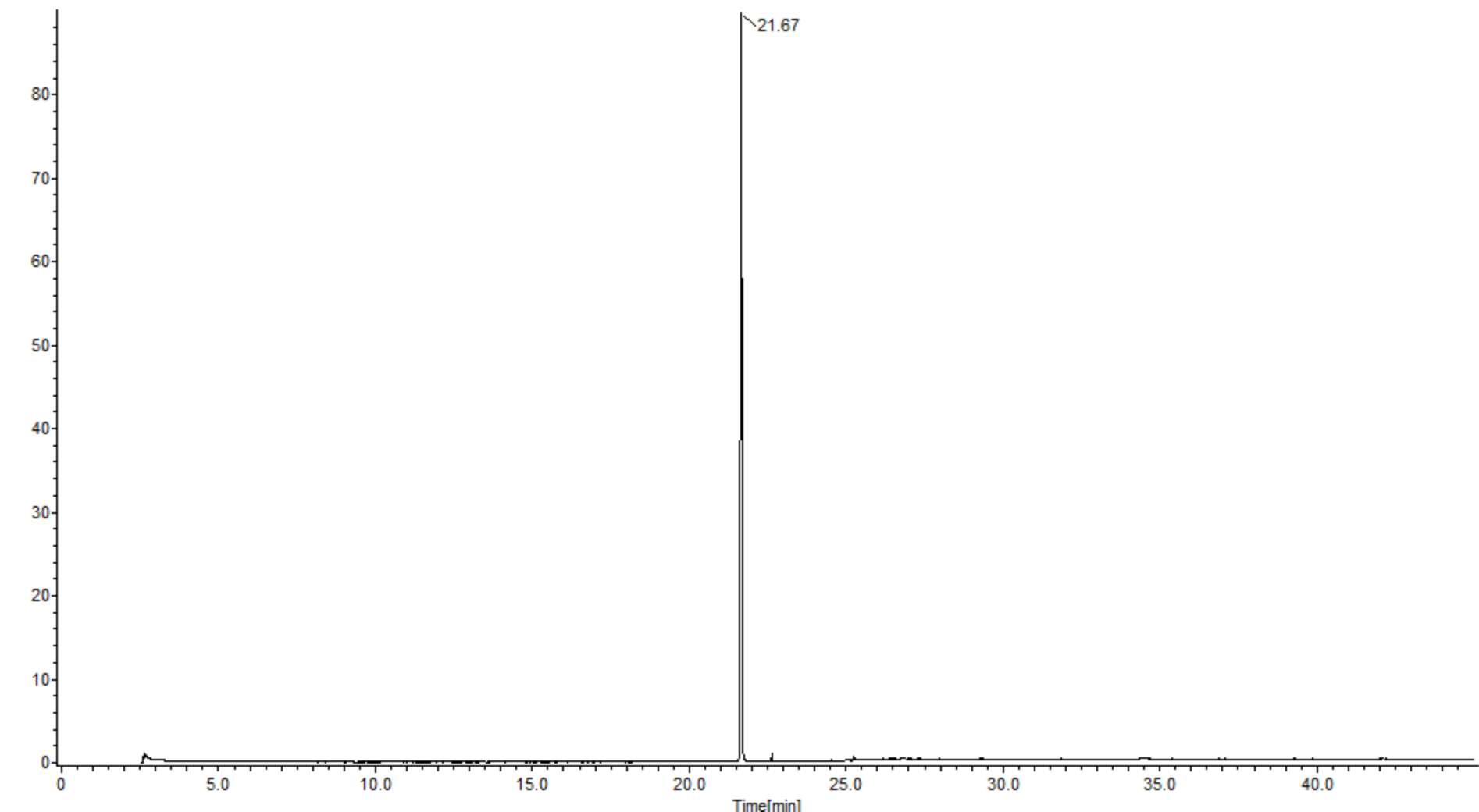

Supplement: Supplementary file 1 [file foods-11-03889-s001.zip › foods-1980133-supplementary/Supplementary data/Figure S1. GC-MS chromatography of compound 3.pdf]

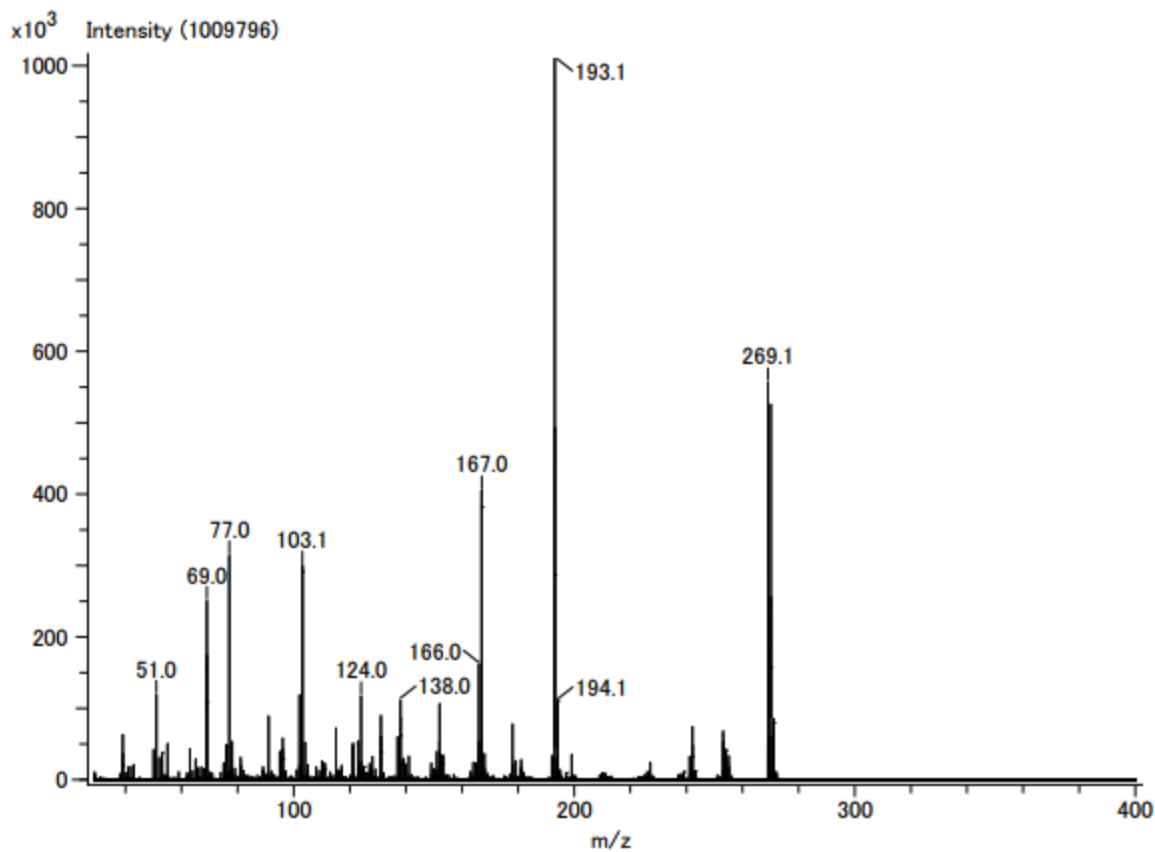

Supplement: Supplementary file 1 [file foods-11-03889-s001.zip › foods-1980133-supplementary/Supplementary data/Figure S10. ESI-MS spectrum of compound 10.pdf]

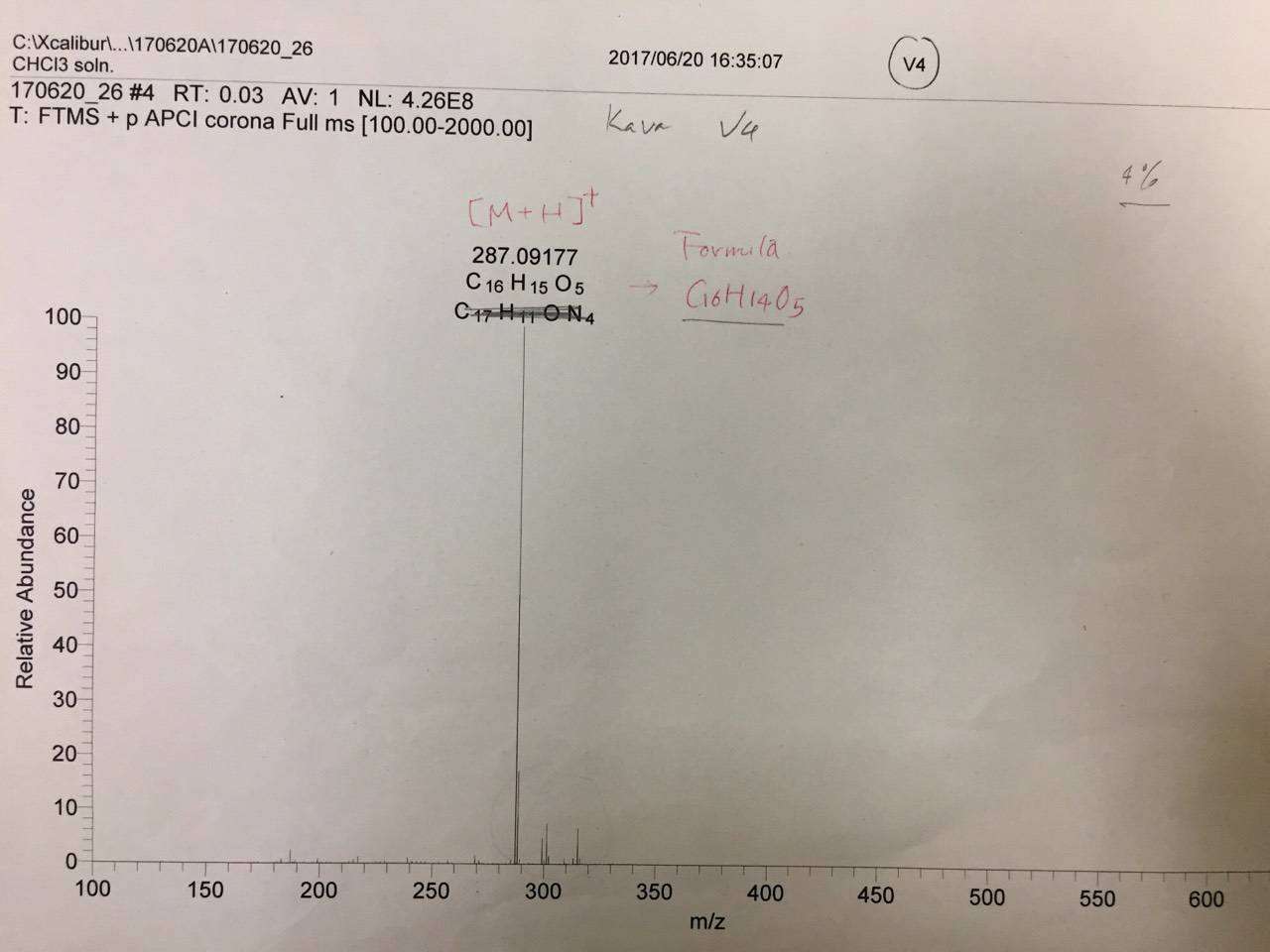

Supplement: Supplementary file 1 [file foods-11-03889-s001.zip › foods-1980133-supplementary/Supplementary data/Figure S4. 1. LC-MS Compound C3.jpg]

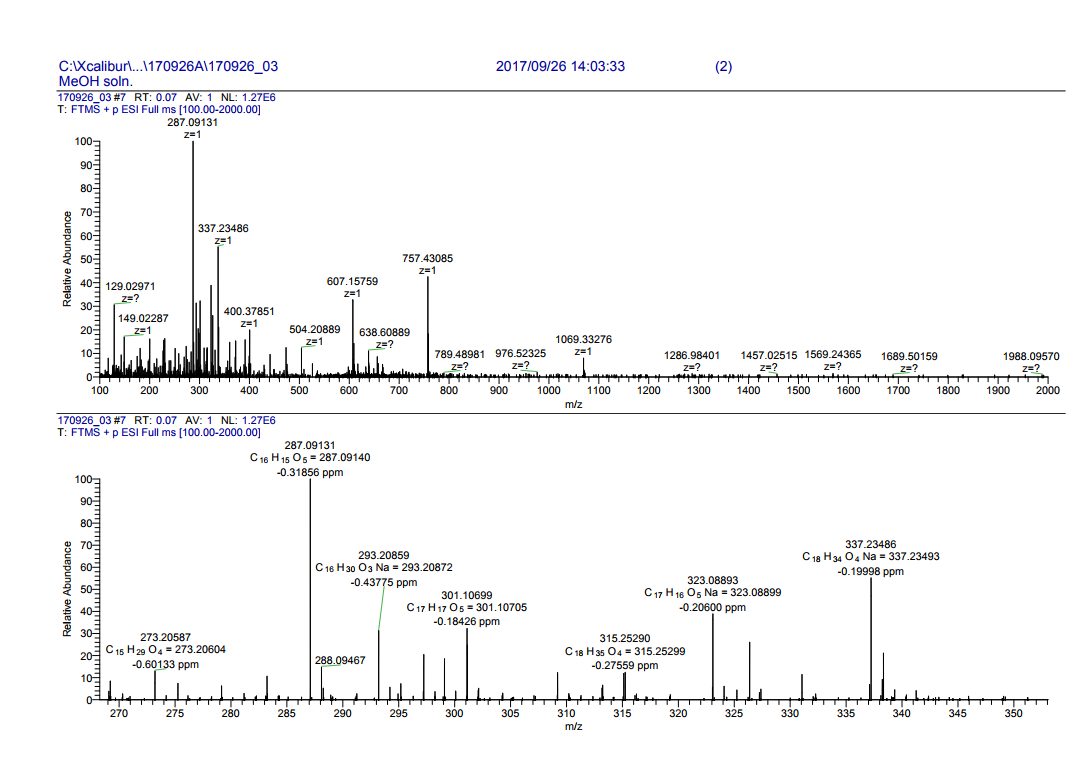

Supplement: Supplementary file 1 [file foods-11-03889-s001.zip › foods-1980133-supplementary/Supplementary data/Figure S4. 2. ESI-MS Compound C3.png]

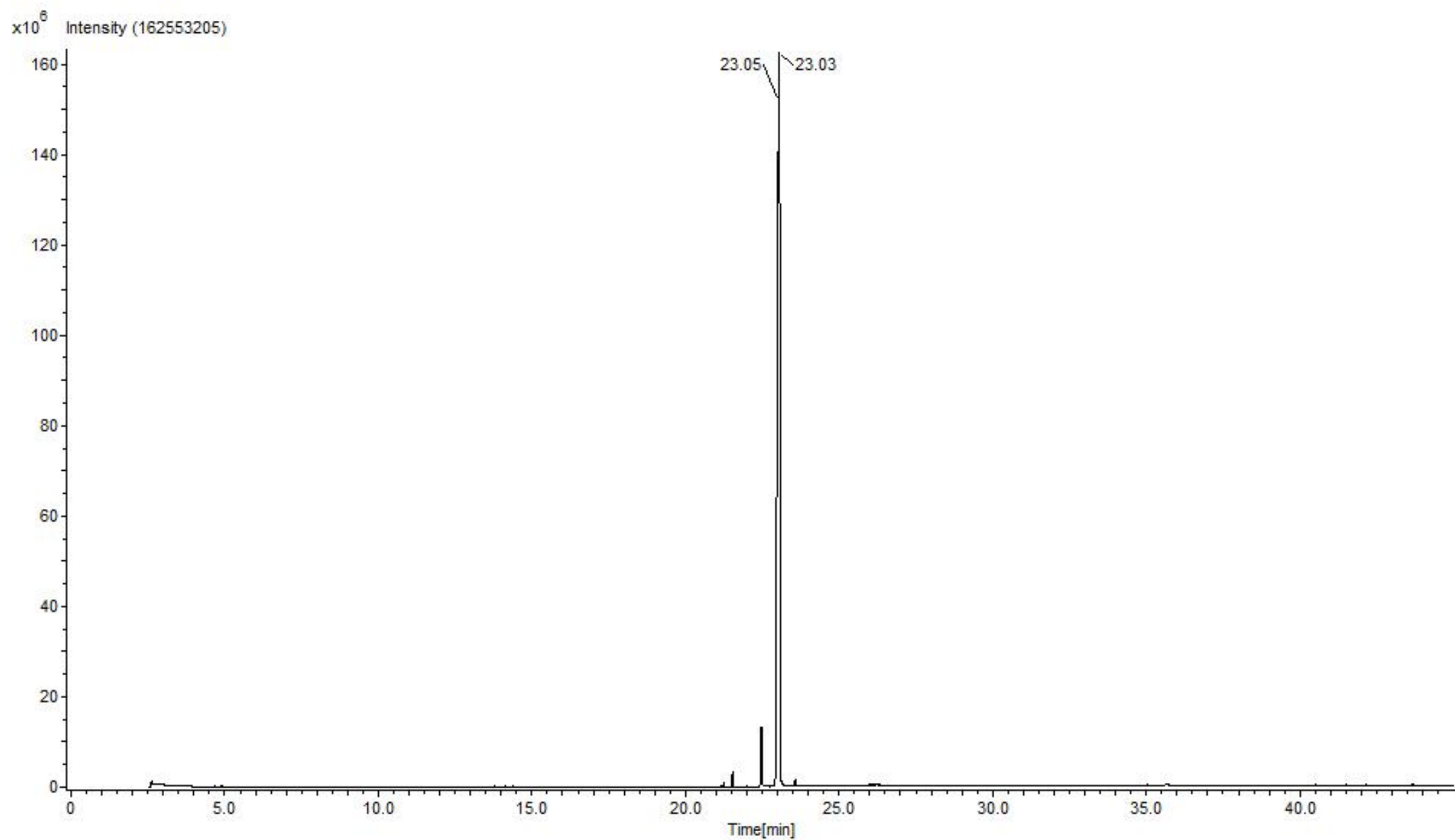

Supplement: Supplementary file 1 [file foods-11-03889-s001.zip › foods-1980133-supplementary/Supplementary data/Figure S5. GC-MS chromatography of compound 4.pdf]

$\times 10^6$  Intensity (124550827)

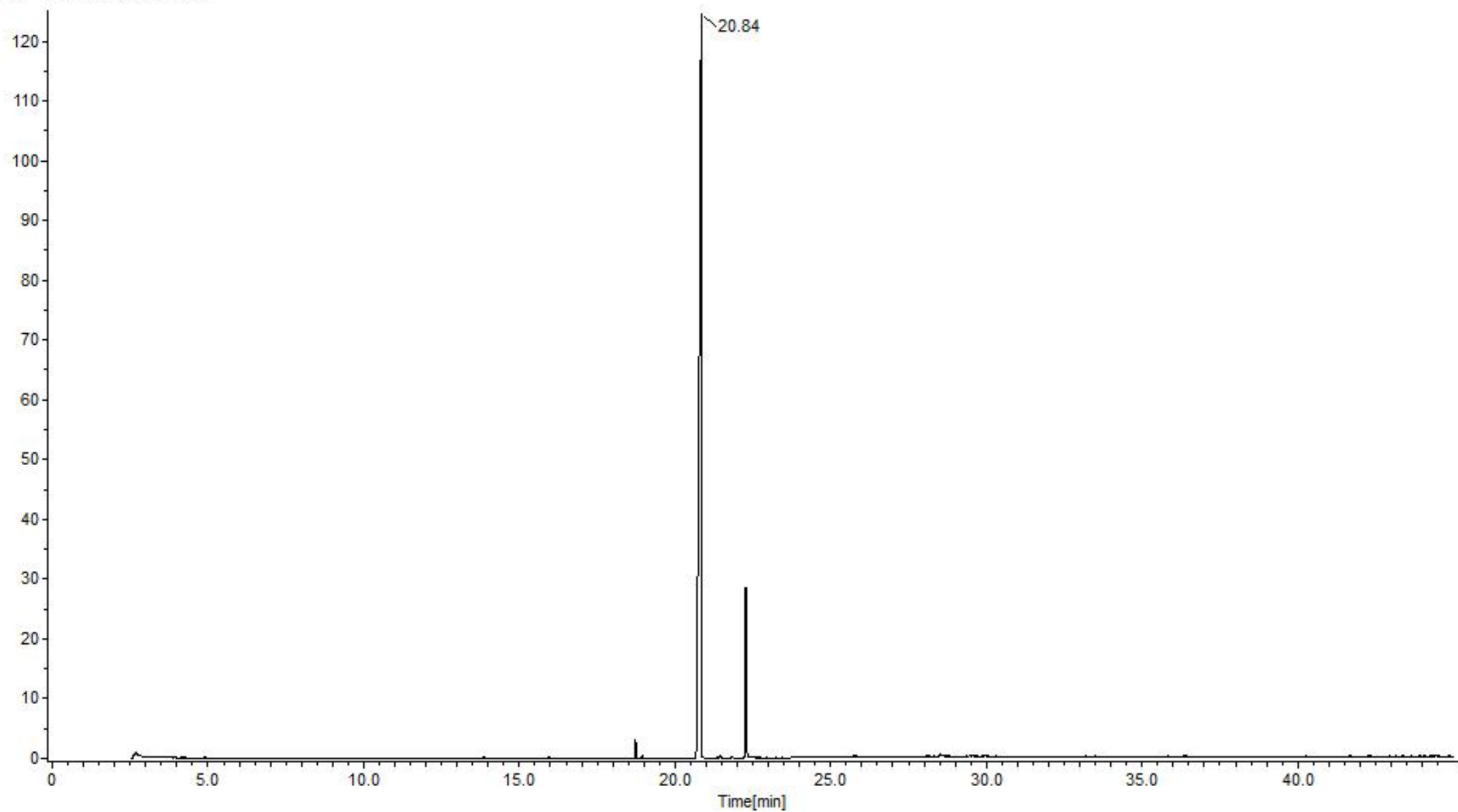

Supplement: Supplementary file 1 [file foods-11-03889-s001.zip › foods-1980133-supplementary/Supplementary data/Figure S7. GC-MS chromatography of compound 5.pdf]
